# Phosphinocyclodextrins as confining units for catalytic metal centres. Applications to carbon–carbon bond forming reactions

**DOI:** 10.3762/bjoc.10.249

**Published:** 2014-10-15

**Authors:** Matthieu Jouffroy, Rafael Gramage-Doria, David Sémeril, Dominique Armspach, Dominique Matt, Werner Oberhauser, Loïc Toupet

**Affiliations:** 1 Laboratoire de Chimie Inorganique Moléculaire et Catalyse, Institut de Chimie UMR 7177 CNRS, Université de Strasbourg, 1, rue Blaise Pascal, 67008 Strasbourg Cedex, France; 2 Istituto di Chimica dei Composti OrganoMetallici CNR, via Madonna del Piano, 10, 50019 Sesto Fiorentino, Firenze, Italy; 3 Groupe Matière Condensée et Matériaux, UMR 6626 CNRS, Université de Rennes 1, 263, avenue du Général Leclerc, 35042 Rennes Cedex, France

**Keywords:** asymmetric hydroformylation, cyclodextrin, Heck reaction, homogeneous catalysis, palladium, phosphine, rhodium

## Abstract

The capacity of two cavity-shaped ligands, HUGPHOS-1 and HUGPHOS-2, to generate exclusively singly phosphorus-ligated complexes, in which the cyclodextrin cavity tightly wraps around the metal centre, was explored with a number of late transition metal cations. Both cyclodextrin-derived ligands were assessed in palladium-catalysed Mizoroki–Heck coupling reactions between aryl bromides and styrene on one hand, and the rhodium-catalysed asymmetric hydroformylation of styrene on the other hand. The inability of both chiral ligands to form standard bis(phosphine) complexes under catalytic conditions was established by high-pressure NMR studies and shown to have a deep impact on the two carbon–carbon bond forming reactions both in terms of activity and selectivity. For example, when used as ligands in the rhodium-catalysed hydroformylation of styrene, they lead to both high isoselectivity and high enantioselectivity. In the study dealing with the Mizoroki–Heck reactions, comparative tests were carried out with WIDEPHOS, a diphosphine analogue of HUGPHOS-2.

## Introduction

**Figure 1:**
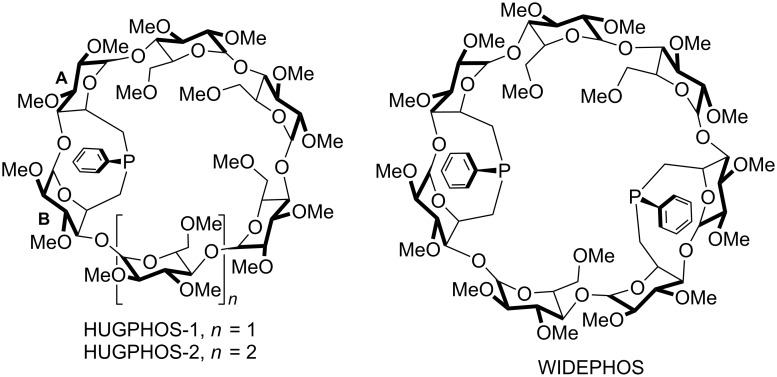
CD-based mono- and diphosphines with inward-pointing phosphorus atoms.

Since the studies of Fu, Buchwald and Hartwig on the use of monophosphine ligands in cross-coupling reactions, notably carbon–carbon ones such as the Mizoroki–Heck [1–3] and Suzuki–Miyaura reactions [4–6], there is a renewed interest for tertiary phosphines that favour the formation of singly phosphorus-ligated complexes when opposed to transition metal ions. Such a behaviour, which was shown to have a deep impact on the catalyst performance, is classically observed with very bulky monophosphines [1,7–10], including dendrimeric ones [11–12], and is also found with hybrid ligands displaying hemilability so as to prevent the coordination of a second phosphorus atom [13–14] or cavity-shaped phosphines [15]. The use of sterically-hindered P(III)-derivatives, notably phosphites [16–21], has also proven beneficial in yet another carbon–carbon forming reaction, namely the rhodium-catalysed hydroformylation [22] of α-olefins [23–29]. By favouring the formation of singly phosphorus-ligated complexes, these ligands not only improve the catalyst activity, but also its regioselectivity, the branched regioisomer(s) being formed at the expense of the linear one. However, when it comes to enantioselectivity, only one chiral mono-P(III) ligand [30–32] has so far shown some potential in the notoriously challenging, but industrially relevant asymmetric hydroformylation [33–37]. Recently, we have synthesised a new type of chiral phosphine ligand (HUGPHOS-1 [38] and HUGPHOS-2 [39], see Figure 1), which consists of methylated cyclodextrins (CD) equipped with an embedded phosphorus atom. In contrast to previously reported monophosphines [40–43] based on methylated CDs [41–44], our ligands have confining properties because of the presence of an inward-pointing P(III) atom [44–45]. The present study is concerned with the ability of HUGPHOS-1 and HUGPHOS-2 to generate exclusively singly P(III)-ligated complexes with a number of *d*^6^ and *d*^8^ metal cations and the evaluation of the catalytic properties of palladium and rhodium complexes of this type in the Mizoroki–Heck and asymmetric hydroformylation reactions. The related trans-chelating diphosphine WIDEPHOS (Figure 1) was also tested for comparison purposes in the case of the Mizoroki–Heck coupling studies.

## Results and Discussion

### Metal coordination

**Scheme 1:**
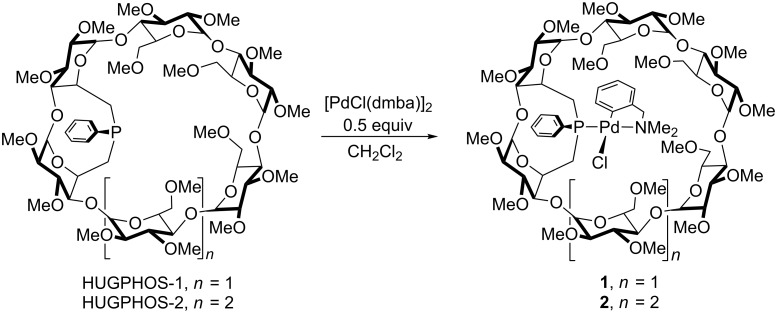
Complexation of a "PdCl(dmba)" unit by HUGPHOS ligands.

As shown previously, HUGPHOS-1 and HUGPHOS-2 are able to accommodate small organometallic moieties, for example the PdCl(dmba) moiety (dmba = Me_2_NCH_2_C_6_H_4_), as in complexes **1** [38] and **2** [45] (Scheme 1). In view of the embracing nature of these cavity-shaped ligands, we wondered whether it would be possible to promote the selective formation of monophosphine complexes with MX_2_ (M = Pd, Pt) fragments that normally form [ML_2_X_2_] complexes with tertiary phosphines.

**Scheme 2:**
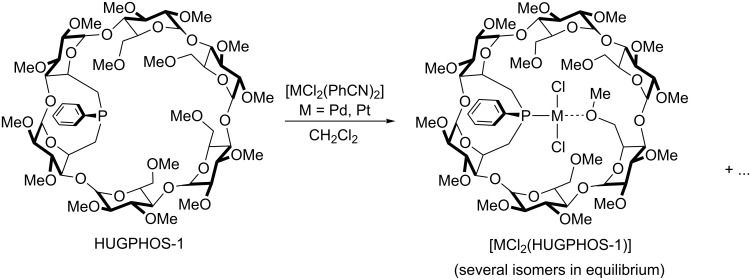
Reaction of HUGPHOS-1 with [MCl_2_(PhCN)_2_] complexes (M = Pd, Pt). Only one isomer with a given MeO–M bond has been drawn.

When reacted with HUGPHOS-1 in CH_2_Cl_2_, both [PdCl_2_(PhCN)_2_] and [PtCl_2_(PhCN)_2_] afforded a mixture of complexes (Scheme 2). The presence of a unique broad signal in each ^31^P{^1^H} NMR spectrum is consistent with the presence of several species in equilibrium. This may reflect exchange processes involving methoxy groups of the primary face and/or free benzonitrile. Mass spectrometric measurements carried out on the crude reaction mixtures showed a peak corresponding to MCl_2_(HUGPHOS-1) fragments. There was no indication for the formation of complexes with a molecular weight higher than that of [MCl_2_(HUGPHOS-1)], this suggesting that no stable bis(phosphine) complexes had formed.

**Scheme 3:**
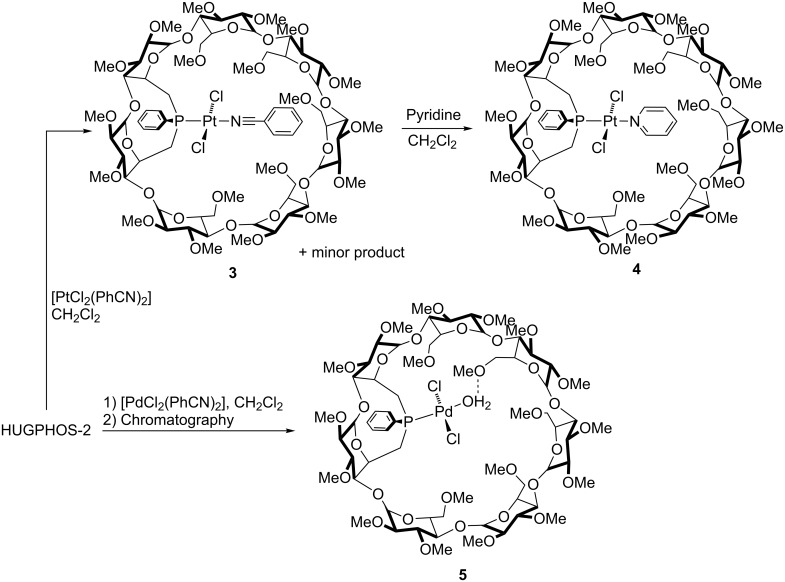
Synthesis of complexes **3**–**5**.

A similar study was carried out with the larger HUGPHOS-2. Its reaction with [PtCl_2_(PhCN)_2_] in CH_2_Cl_2_ resulted in the formation of the monophosphine complex [PtCl_2_(HUGPHOS-2)(PhCN)] (**3**) in 95% yield, but this complex could not be separated from a minor product, probably the benzonitrile-free complex [PtCl_2_(HUGPHOS-2)]. The ^31^P{^1^H} NMR spectrum of **3** showed a sharp signal at 3.2 ppm, with Pt satellites (^1^*J*_P,Pt_ = 3433 Hz). The mass spectrum of the mixture of products showed an intense peak at *m*/*z* = 1866.61 (100%), corresponding to the [M + Na]^+^ cation, as well as a peak resulting from the loss of PhCN (*m*/*z* = 1763.57 (11) [M – PhCN + Na]^+^). No peaks corresponding to compounds with two phosphine ligands were detected in the spectrum. Addition of 1 equiv of pyridine to the mixture containing **3** gave quantitatively complex **4** (Scheme 3). The ^1^H NMR spectrum of **4** shows that some H-5 signals are significantly low-field shifted with respect to their counterparts in the free ligand, an observation which is indicative of an entrapped chlorido ligand. Note that the marked affinity of CDs for metal halide bonds is well documented [46–47]. The trans *P*,*N* configuration was deduced from a ROESY experiment which showed strong correlations between the pyridinic H-4 proton and some inner cavity H-5 protons. Also, a ^1^*J*_P,Pt_ coupling constant typical of this particular geometry (^1^*J*_P,Pt_ = 3542 Hz) unequivocally established the configuration of the complex [48].

**Figure 2:**
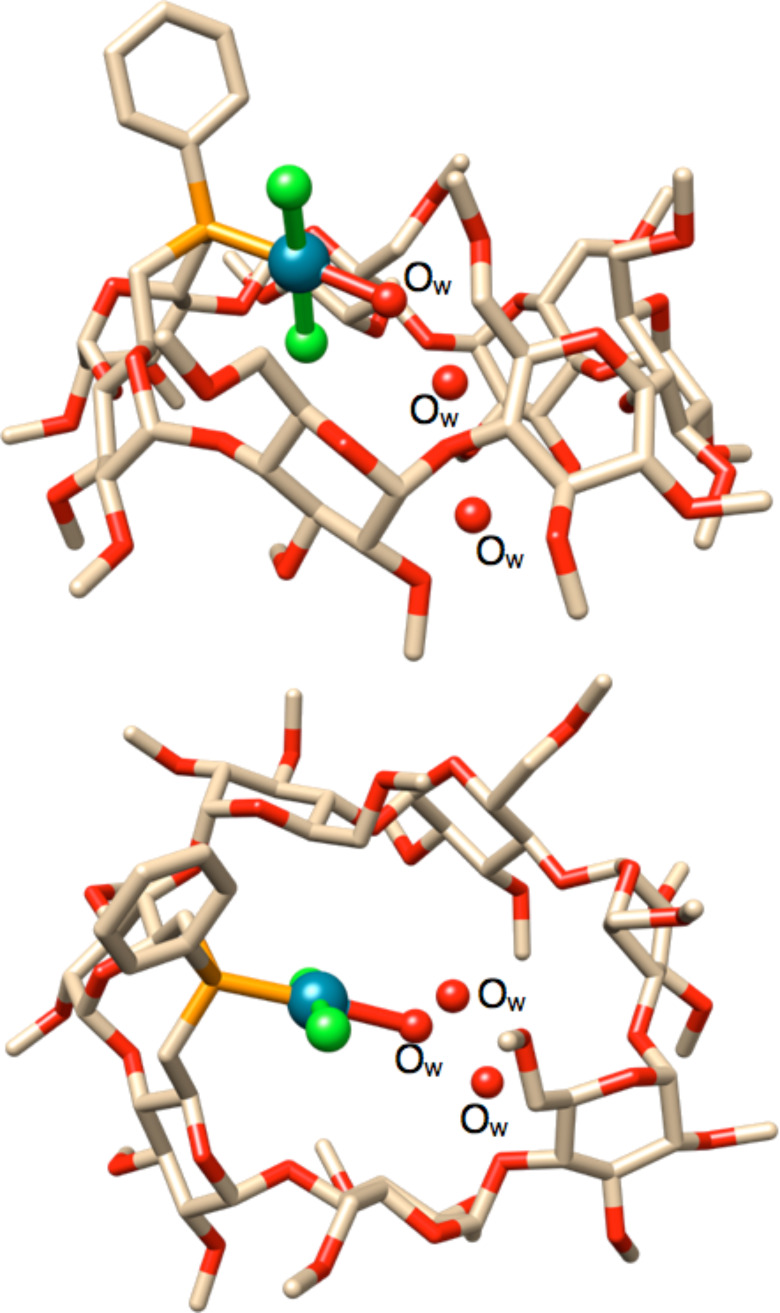
X-ray structure of aqua palladium complex **5** [44] (top: side view; bottom: view from the primary face). The cavity contains two non-coordinated water molecules. O_w_ stands for water molecules.

It should be reminded that monophosphine complexes of the general formula [MX_2_(phosphine)(pyridine)] usually undergo facile ligand dissociation in solution [48]. This is, however, not the case for complex **4**. Owing to the protecting role played by the cavity, this complex proved to be particularly robust, to such an extent that it could be purified by column chromatography (this being necessary for removing PhCN) without noticeable decomposition. Attempts to produce a palladium analogue of **3** starting from [PdCl_2_(PhCN)_2_] failed, the corresponding reaction leading to a mixture of equilibrating species that could not be separated. However, when the reaction mixture was subjected to column chromatography on wet SiO_2_, a single aqua palladium complex (**5**) was recovered in high yield (90%). The P-monoligated nature of this complex was inferred from its mass spectrum, which displays a strong peak at *m*/*z* = 1675.52 corresponding to the [M + Li]^+^ ion. The ^31^P{^1^H} NMR spectrum of **5** revealed a single, slightly broad singlet at δ = 34.4 ppm. Although not visible at room temperature, the coordinated water molecule appeared as a broad singlet at δ = 5.64 ppm in the ^1^H NMR spectrum recorded at −80 °C [45]. This chemical shift value is typical for aqua palladium complexes [49–51]. A single crystal X-ray diffraction study (Figure 2) confirmed the coordination of a {PdCl_2_(H_2_O)} fragment, which lies inside the β-CD cavity. To date, only one other example of [MCl_2_(phosphine)(H_2_O)] aqua complex has been reported [52].

**Scheme 4:**
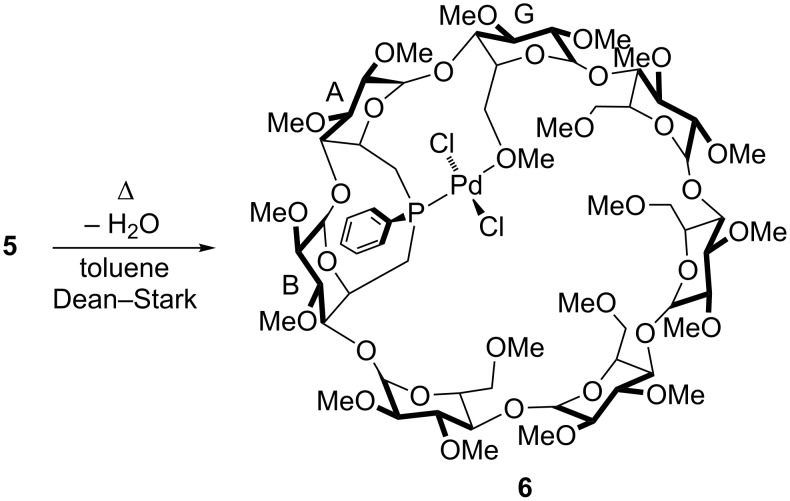
Dehydration of Pd(II) complex **5**.

Complex **5** could be dehydrated using a Dean–Stark apparatus to give the corresponding methoxy-bonded complex **6** (Scheme 4). The mass spectrum of **6** shows a strong peak at *m*/*z* = 1673.52 having the isotopic profile expected for the [M + Na]^+^ ion. The ^1^H NMR spectra of **5** and **6** are very similar, however small differences could be detected, in particular in the chemical shift range where methoxy protons resonate. Although it was not possible to determine which methoxy group was bonded to the metal centre because of overlapping signals, coordination of the one belonging to glucose unit G seems to be the most likely according to CPK models. Also, the anomeric protons of **6** lie in a wider range (Δδ = 0.36 ppm) than those of less distorted **5** (Δδ = 0.18 ppm). Note that complex **5** is readily reformed in the presence of water.

**Figure 3:**
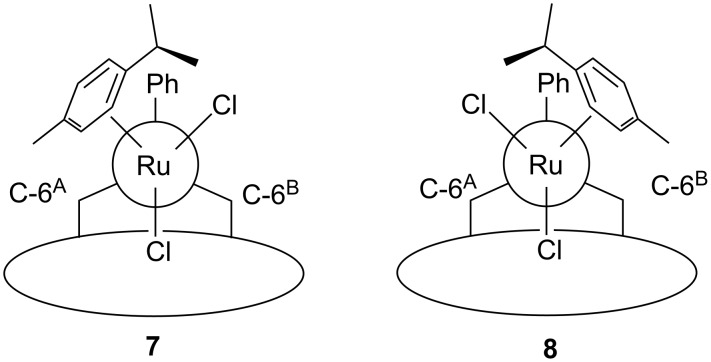
Ruthenium complexes **7** and **8** in Newman projection along the Ru–P bond.

HUGPHOS-2 was further opposed to [RuCl_2_(*p*-cymene)]_2_. This reaction gave a 57:43 mixture of the two rotamers **7** and **8**, which could be separated by column chromatography (Figure 3). Careful examination of the ROESY spectrum of **7** indicates that the rotations about the P–Ru bond and the Ru–arene bond are both restricted. Thus, the ROESY spectrum of this complex showed correlations between the Me group of the *p-*cymene ligand and protons belonging to glucose units G and A, but not with protons from the PPh ring or glucose units B and C. In keeping with these findings, the only cross peaks associated with the C*H*Me_2_ proton of **7** involved protons from the PPh ring. Similar observations, which establish the same blocked rotations about the P–Ru and the Ru–arene bonds were made for **8**. It should be emphasised that prolonged heating of **7** in refluxing toluene did not result in the formation of **8**. Restricted rotation about the Ru–P bond in **7** is possibly caused by the entrapment of one of the chlorido ligands inside the cavity.

**Figure 4:**
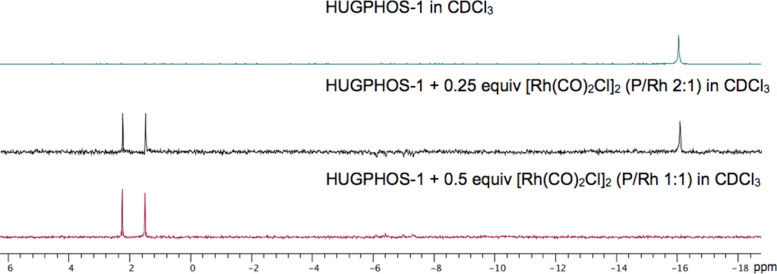
Titration of HUGPHOS-1 with [Rh(CO)_2_Cl]_2_ at 25 °C.

**Scheme 5:**
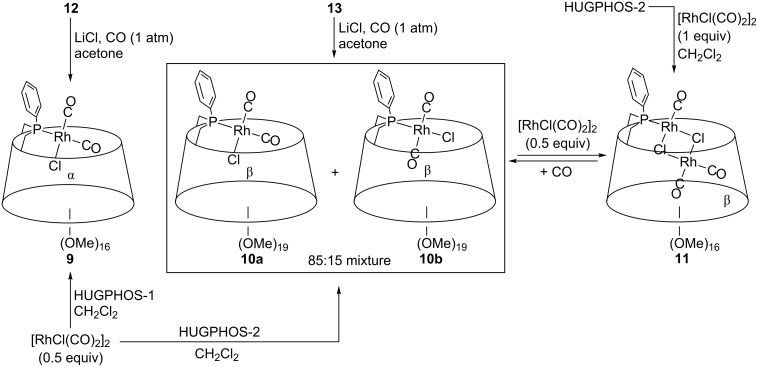
Synthesis of rhodium carbonyl complexes **9**–**11**.

Further proof for the capacity of HUGPHOS-1 to prevent the formation of bis(phosphine) complexes came from the reaction of [RhCl(CO)_2_]_2_ with excess ligand, which only produced *cis*-[RhCl(HUGPHOS-1)(CO)_2_] (**9**) together with free phosphine, rather than the expected complex *trans*-[RhCl(HUGPHOS-1)_2_(CO)] (Figure 4, Scheme 5). The corresponding IR spectrum is typical of CO ligands in relative *cis* positions (strong CO bands at 2009 and 2082 cm^−1^). Further, with some CD H-5 protons belonging to non-bridged glucose units strongly upfield shifted upon metal complexation (Δδ up to 0.7 ppm), the ^1^H NMR spectrum of **9** is fully consistent with a CD-encapsulated chlorido ligand [53], which can only mean that two *cis* configured CO ligands are present [22].

**Scheme 6:**
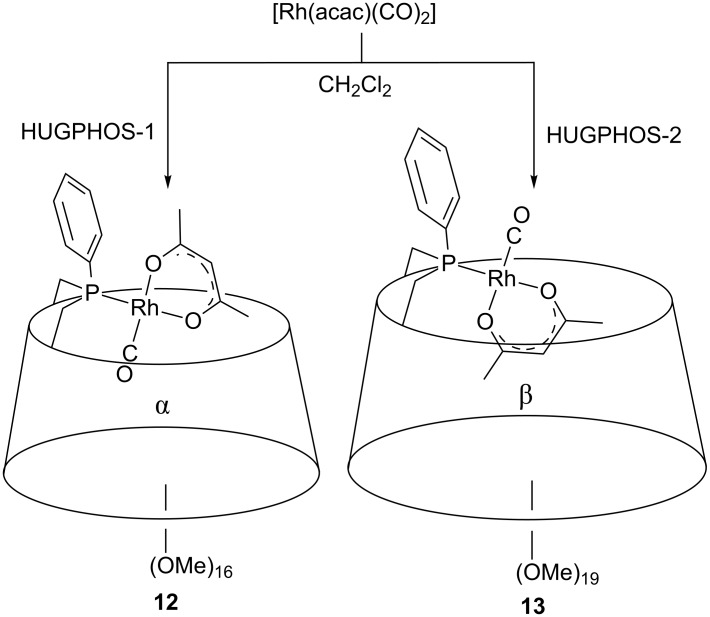
Synthesis of rhodium complexes **12** and **13**.

In the case of HUGPHOS-2, the reaction with 0.5 equiv of [RhCl(CO)_2_]_2_ resulted in the formation of an 85:15 mixture of the inseparable, mononuclear stereoisomeric complexes **10a** and **10b** (Scheme 5), in which the two CO ligands are respectively *cis* (strong CO IR bands at 2009 and 2082 cm^−1^ [54]) and *trans* (strong CO IR band at 1985 cm^−1^ [55–56]) configured. Remarkably, the same ratio of stereoisomers was obtained when rhodium complex **13** (see Scheme 6) was treated with LiCl under CO (1 atm). When the reaction between HUGPHOS-2 and [RhCl(CO)_2_]_2_ was repeated, but applying a 1:1 instead of 1:0.5 stoichiometry, the dinuclear complex [Rh_2_(*μ*-Cl)_2_(HUGPHOS-2)(CO)_3_] (**11**) formed (see Experimental part). Clearly, the cavity of HUGPHOS-2 is capable of accommodating up to two metal centres, whereas the smaller HUGPHOS-1 ligand is unable to do so.

The HUGPHOS ligands were further reacted with [Rh(acac)(CO)_2_] (acac = acetylacetonate), this producing quantitatively the singly P-ligated rhodium complexes **12** and **13** (Scheme 6). While in **13** the large β-CD cavity hosts the acac ligand, the same ligand is located outside the α-CD cavity in **12** according to ROESY experiments. On the other hand, the smaller CO rod is nested in the α-CD cavity of **12**, and located outside the β-CD cavity in **13**. Clearly, size selectivity is at work in these metal complexes. As already observed for complex **4**, both **12** and **13** are remarkably stable and can be purified by column chromatography on SiO_2_. This makes them, a priori, good candidates for hydroformylation studies.

### High-pressure NMR studies

**Scheme 7:**
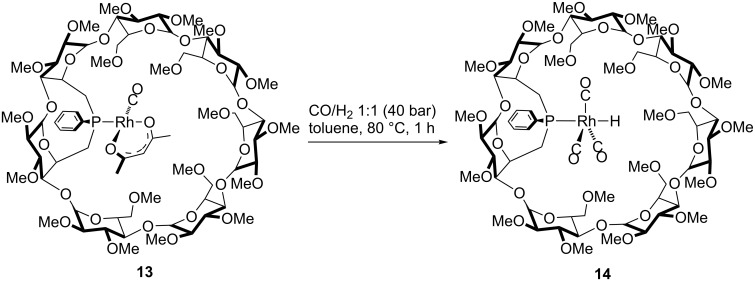
Selective formation of complex **14** under 40 bar CO/H_2_ at 80 °C.

Upon subjecting complex **13** to a syngas (1:1 CO/H_2_ mixture) pressure of 40 bar at 80 °C in toluene-*d*_8_ (Scheme 7)_,_ the only species that was detected by high-pressure NMR and IR spectroscopy [44] was complex *trans*-[RhH(HUGPHOS-2)(CO)_3_] (**14**). Confirmation of a 5-coordinate rhodium centre in **14** came from MS measurements carried out from the toluene-*d*_8_ solution, which showed the presence of a peak at 1663.53 (1%, exact isotopic profile) corresponding to the expected [M + H]^+^ ion.

**Figure 5:**
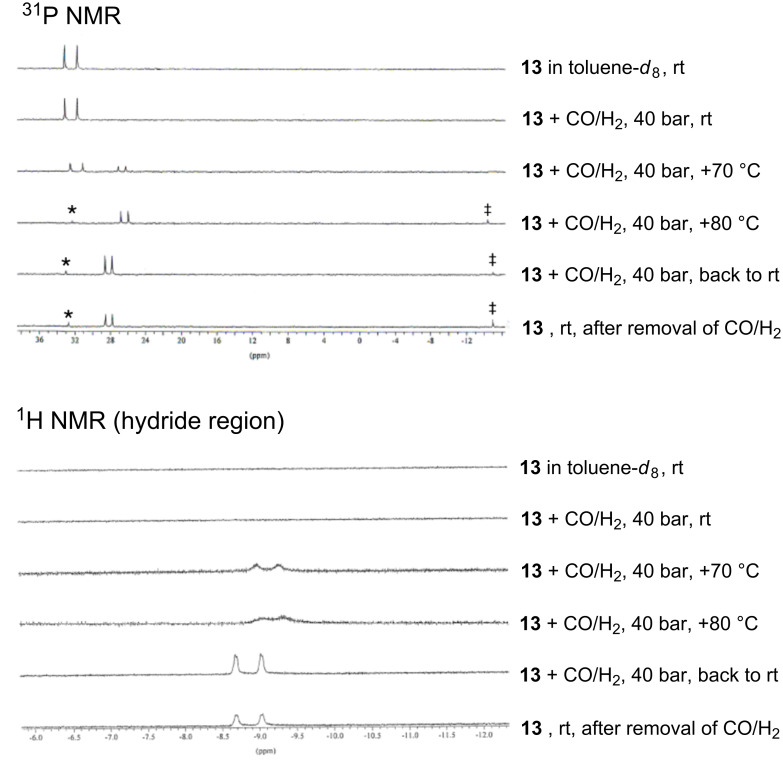
High pressure NMR spectra of **13** under CO/H_2_ (1:1) recorded in toluene-*d*_8_ (at various temperatures and pressures), showing its conversion into *trans*-[RhH(CO)_3_(HUGPHOS-2)] (**14**). The asterisk and double cross denote traces of oxidized and free HUGPHOS-2, respectively.

**Figure 6:**
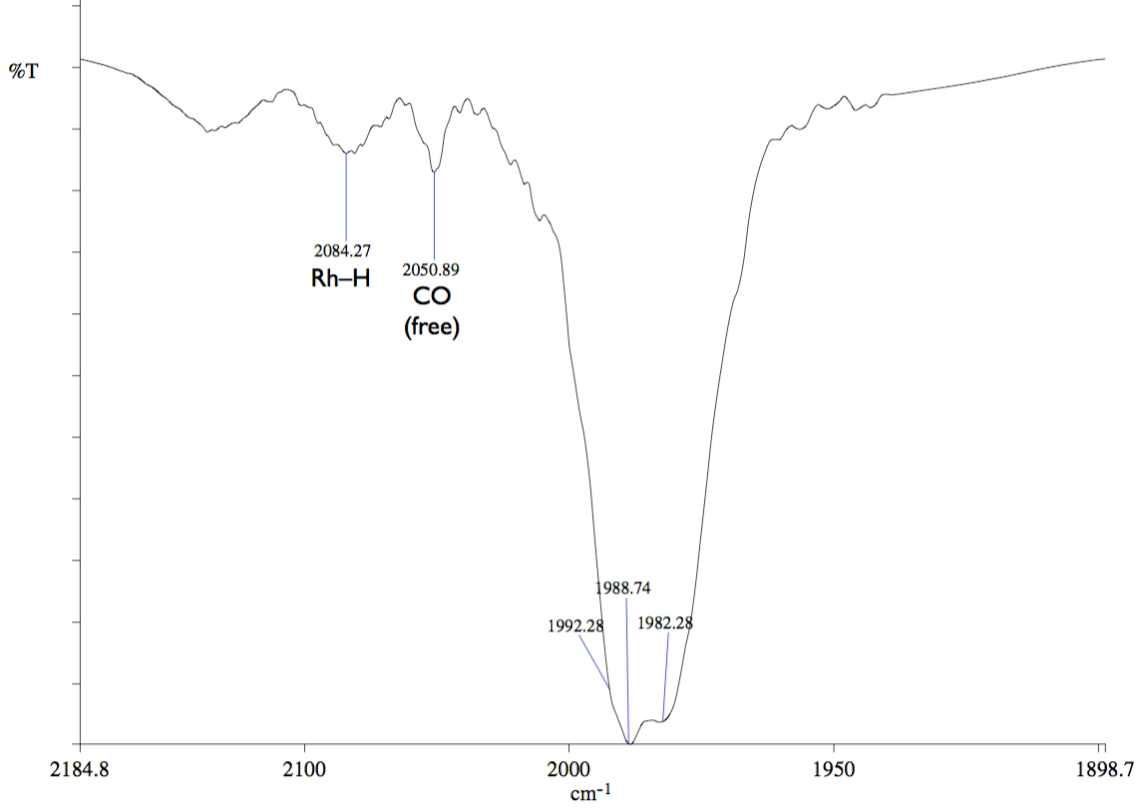
IR spectra of **14** recorded in CH_2_Cl_2_ at 50 °C under 40 bar of CO/H_2_ 1:1.

The ^31^P{^1^H} NMR spectrum of **14** consisted of a doublet at 28.1 ppm (^1^*J*_P,Rh_ = 95 Hz). The presence of a hydride ligand trans to the P atom was inferred from the ^1^H NMR spectrum of **14** (25 °C, 40 bar), which displays a signal at –8.8 ppm (^1^*J*_H,Rh_ = 6.2 Hz) with a large ^2^*J*(H,P) coupling constant (^2^*J*_H,P_ = 103 Hz) (Figure 5). The three close together carbonyl bands at 1982 (vs), 1989 (vs), and 1992 (sh, vs) cm^–1^ and the additional Rh–H broad band of low intensity at 2084 cm^−1^ (Figure 6) in the IR spectrum of **14** measured at 50 °C under 40 bar of CO/H_2_ are consistent with a trigonal bipyramidal complex having *C*_1_ symmetry.

The carbonyl region of the IR spectrum of **13** markedly differs from that of the only other reported *trans*-[RhH(CO)_3_L] complex (where L is a bulky phosphoramidite), the observed three carbonyl bands (2055 (sh), 2022 (w) and 1998 (s) cm^−1^) being here spread over a larger frequency range [31]. Note that the related cobalt complex *trans*-[CoH(CO)_3_(PCy_3_)] displays a higher symmetry (*D*_3h_), and accordingly, its IR spectrum shows only one carbonyl band [57–60].

**Figure 7:**
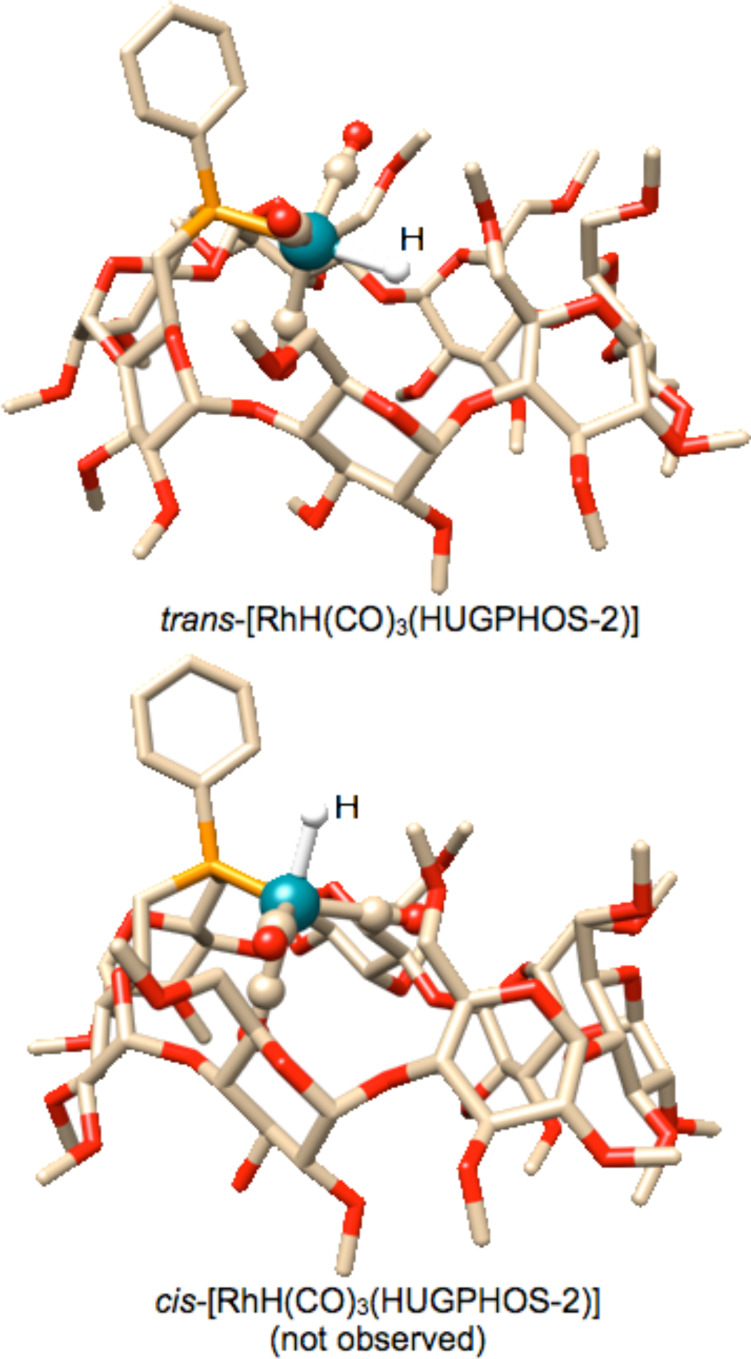
Calculated structures (Spartan 10) of trigonal bipyramidal [RhH(CO)_3_(HUGPHOS-2)] with the phosphorus being either in apical (top) or equatorial (bottom) position.

The phosphorus atom in **14** probably binds the rhodium centre specifically in an apical fashion because of the confining properties of the phosphine. In fact, the trigonal bipyramidal complex has no other option, but to adopt a linear P–Rh–H arrangement (Figure 7), so that steric interactions between the carbonyl ligands and the cavity inner wall are reduced to the maximum.

### Hydroformylation of styrene

The results of the above HP-NMR studies prompted us to investigate the properties of HUGPHOS ligands in an asymmetric hydroformylation [33–37]. Styrene was chosen as this substrate is compatible in terms of size with both CD cavities. Five different parameters, namely temperature, pressure, CO/H_2_ and L/Rh ratios as well as pre-catalyst loadings were varied during the catalytic study (Table 1 and Table 2). When standard hydroformylation conditions (Table 1, entry 1) were applied, next to full conversion was observed after 24 h with **13**. As expected, the branched product was formed predominantly, however with poor enantioselectivity. Surprisingly, raising the CO/H_2_ from 1:1 to 1:2, which is known to speed up the reaction, produced the opposite effect and was also detrimental to enantioselectivity [22], but without significantly altering regioselectivity (Table 1, entry 2). On the other hand, increasing the partial CO pressure led to a marked reaction rate increase, however with neither enantioselectivity, nor regioselectivity increase (Table 1, entry 3). An unexpected observation was that addition of free ligand to the reaction medium maintained at 80 °C (20 bar) was detrimental to the catalyst activity, this suggesting that at this temperature unreactive bis(phosphine) complexes formed (Table 1, entries 4 and 5), although for HUGPHOS ligands bis(phosphine) complexes have never been isolated so far.

**Table 1:** Rhodium-catalysed hydroformylation of styrene using precatalyst **13** – variation of ligand/Rh and CO/H_2_ ratio.^a^

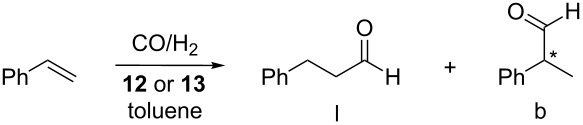							

entry	equiv of HUGPHOS-2^b^	CO/H_2_ ratio	conv^c^	aldehydes^c^		b:l^d^	ee^e^
			[%]	l [%]	b [%]		[%]

1	0	1/1	96.8	37.1	62.9	1.7	27 (*R*)
2	0	1/2	71.5	32.0	68.0	2.2	17 (*R*)
3	0	2/1	99.3	32.6	67.4	2.1	26 (*R*)
4	1	1/1	75.1	38.2	61.8	1.6	19 (*R*)
5	4	1/1	12.9	20.3	79.7	3.9	36 (*R*)

^a^Styrene (5 mmol), styrene/complex = 2500, *T* = 80 °C, *t* = 24 h, *P*(CO/H_2_) = 20 bar, toluene/*n*-decane (15 mL/0.5 mL), incubation overnight at 80 °C under *P*(CO/H_2_) = 20 bar. ^b^Equiv of free ligand HUGPHOS-2 added to preformed rhodium complex **13** after overnight incubation. ^c^Determined by GC using decane as internal standard. ^d^b:l (branched:linear) aldehyde ratio. ^e^Determined by chiral-phase GC after reduction with LiAlH_4_.

Raising the temperature to 120 °C caused the catalyst activity to drop significantly and led predominantly to the (*S*)-enantiomer, suggesting a profound transformation of the catalyst upon heating (Table 2, entry 2). However, both regioselectivity and enantioselectivity improved significantly upon lowering the temperature, reaching 63% ee at 60 °C (Table 2, entry 3). Increasing the metal to substrate ratio by 10-fold and further lowering the temperature allowed to maintain a reasonable activity while further increasing the ee value and b:l ratio (Table 2, entry 4). Interestingly, complexes **12** and **13** led roughly to the same results (Table 2, entries 5 and 12). This means that the reaction is insensitive to cavity size, this being indicative of a catalytic transformation taking place at the cavity entrance, rather than inside. Pressure had also a dramatic effect on both regioselectivity and enantioselectivity as raising it from 5 to 40 bar increased the ee value by a staggering 49% and the b:l ratio from 3.7 to 24.6 (Table 2, entries 10 and 11). Not surprisingly, the best result (Table 2, entry 12) was obtained at room temperature and high pressure, the ee value and the proportion of branched aldehyde reaching then 95% and 98.3%, respectively.

**Table 2:** Rhodium-catalysed hydroformylation of styrene using precatalysts **12** and **13** – variation of pressure and temperature.^a^

entry	complex	*P*(CO/H_2_)^b^	*T*	conv^c^	aldehydes^c^		b/l^d^	ee^e^
		[bar]	[°C]	[%]	l [%]	b [%]		[%]

1	**13**	20	80	96.8	37.1	62.9	1.7	27 (*R*)
2	**13**	20	120	31.5	43.0	57.0	1.3	34 (*S*)
3	**13**	20	60	43.7	13.9	86.1	6.2	63 (*R*)
4^f^	**13**	20	40	79.0	6.8	93.2	13.7	80 (*R*)
5^f^	**13**	40	20	66.2	1.7	98.3	57.8	92 (*R*)

6	**12**	20	80	86.3	27.2	72.8	2.7	33 (*R*)
7^f^	**12**	20	60	100	11.4	88.6	7.8	62 (*R*)
8^f^	**12**	20	40	99.8	6.3	93.7	14.9	80 (*R*)
9^f^	**12**	20	20	30.6	1.0	99.0	99.0	93 (*R*)

10^f^	**12**	5	40	19.8	21.4	78.6	3.7	41 (*R*)
11^f^	**12**	40	40	99.2	3.9	96.1	24.6	90 (*R*)
12^f^	**12**	40	20	60.7	1.7	98.3	57.8	95 (*R*)
13^f^	**12**	40	4	34.0	traces	100	>100^g^	93 (*R*)

^a^Styrene (5 mmol), styrene/complex = 2500, *t* = 24 h, toluene/*n*-decane (15 mL/0.5 mL), incubation overnight at 80 °C under *P*(CO/H_2_) = 20 bar. ^b^CO/H_2_ 1:1 v/v. ^c^Determined by GC using decane as internal standard. ^d^b:l aldehyde ratio. ^e^Determined by chiral-phase GC after reduction with LiAlH_4_.^f^Run carried out with a ratio styrene/complex = 250. ^g^Exact value not determined because of a very low amount of linear aldehydes.

**Scheme 8:**
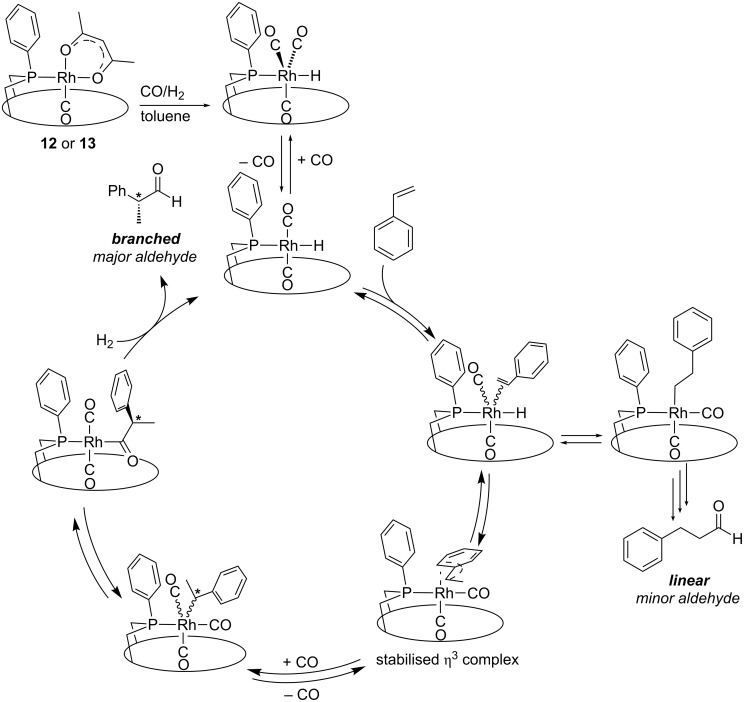
Possible mechanism for the hydroformylation of styrene when using monophosphine complexes **12** or **13** as precatalysts.

Clearly, isoregioselectivity increases concomitantly with enantioselectivity contrary to what is generally observed [37], probably because the singly phosphine-ligated active species behaves differently from the usual bis(phosphine) complexes (Scheme 8). The observed enantio- and isoregioselectivities are amongst the highest reported for the asymmetric hydroformylation of styrene [61–63]. The presence of monophosphine intermediates (and not bis(phosphine) ones) in the catalytic cycle is likely to favour the formation of a [Rh(η^3^-(styrenyl))(CO)_2_] intermediate [31–32,37], precursor of the branched aldehyde, over that of the electron poorer [Rh(σ-(phenylethyl))(CO)_2_] isomer, which leads to the linear aldehyde. The high enantioselectivities probably arise from the embracing properties of the HUGPHOS ligands, which facilitates chirality transfer.

### Heck cross-coupling

Phosphine-assisted Heck reactions strongly depend on the bulkiness of the phosphine used [64]. To assess HUGPHOS-2 in Heck coupling, we focused on the reaction between styrene and aryl bromides using [Pd(OAc)_2_] (OAc = acetate) as a palladium source [65]. In a preliminary study, 4-bromoanisole was reacted for 1 h with styrene in *N*,*N*-dimethylformamide (DMF) in the presence of Cs_2_CO_3_ (Table 3). The runs were carried out at different temperatures and L:M ratios. The highest yield (46.5%) was obtained when operating with 1 mol-% catalyst and one equivalent of HUGPHOS-2 per palladium (Table 3, entry 5). Higher phosphine:Pd ratios did not improve the catalytic outcome (Table 3, entry 4). These results clearly indicate that a single phosphine ligand is sufficient to stabilise the active palladium species. We observed that by raising the temperature to 130 °C, the conversions remained practically unchanged (Table 3, entry 6). For comparison purposes, we also assessed the catalytic behaviour of the related diphosphine WIDEPHOS [39,52]. When using a WIDEPHOS:Pd ratio of 1:1, practically no reaction occurred (Table 3, entry 5). This is probably caused by the formation of a stable pseudo-*trans*-chelate complex with this ligand, which forbids completion of the catalytic cycle [66–67]. Clearly, decoordination of a phosphine end cannot take place in this stable *trans* complex, which seems vital for completion of the whole catalytic cycle [68]. However, when applying a WIDEPHOS:Pd ratio of 1:2, the reaction proceeded with 10.4% conversion (Table 3, entry 2). With the same ratio and by raising the temperature to 130 °C, the conversion increased to 20.7% (Table 3, entry 3). These results suggest that in the presence of an excess palladium, WIDEPHOS operates as a ligand with two independent monophosphine arms, each of them binding a palladium atom.

**Table 3:** Optimisation of conditions for the Heck cross-coupling of 4-bromoanisole with styrene using HUGPHOS-2 as ligand.^a^

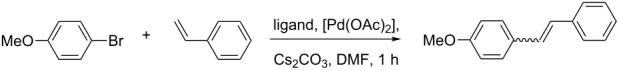				

entry	ligand:[Pd(OAc)_2_] ratio	*T* [°C]	conv [%]^b^	
			HUGPHOS-2	WIDEPHOS

1	0:1	110	4.3	5.6
2	1:2	110	17.6	10.4
3	1:2	130	22.4	20.7
4	2:1	110	45.7	/
5	1:1	110	46.5	<1
6	1:1	130	45.5	/

^a^[Pd(OAc)_2_] (5 × 10^−3^ mmol), 4-bromoanisole (0.5 mmol), styrene (1.0 mmol), Cs_2_CO_3_ (1.0 mmol), DMF (1.5 mL), decane (0.05 mL), 1 h. ^b^Conversions were determined by GC using decane as internal standard.

We then applied the aforementioned optimal conditions in the coupling of styrene with different aryl bromides (Table 4), using either HUGPHOS-(1 or 2) or WIDEPHOS as ligands. In the case of HUGPHOS-2, a conversion of 61.0% was observed for 4-bromotoluene, while the 3- and 2-substituted isomers led to conversions of 37.9% and 28.6%, respectively (Table 4, entries 2–4). Not surprisingly, the activated 2-bromo-6-methoxynaphtalene afforded the corresponding coupling product in relatively high yield (71%; Table 4, entry 5). As already observed in the hydroformylation experiments, HUGPHOS-1 generally gave slightly better results than the larger HUGPHOS-2, which points to a catalytic reaction taking place outside the cavity. It is noteworthy that the activities obtained in Mizoroki–Heck coupling with the above catalytic systems lie in the range obtained with other phosphines [69–70], these being, however, always used in excess [2,71]. In fact, HUGPHOS-derived catalysts are much more active than previously reported CD-based Heck coupling catalysts [72]. The relatively low performance of WIDEPHOS, when operating as a bis(monodentate) ligand, is probably the result of the severe steric encumbrance generated within the cavity of the postulated dinuclear complex.

**Table 4:** Palladium-catalysed Mizoroki–Heck cross-coupling of arylbromides with styrene using HUGPHOS-1, HUGPHOS-2 and WIDEPHOS.^a^

entry	ArBr	conv [%]^b^		
		HUGPHOS-1	HUGPHOS-2	WIDEPHOS

1	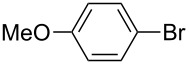	58.3	45.5	20.7
2	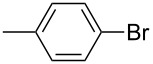	50.5	61.0	31.1
3	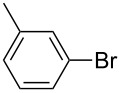	42.4	37.9	25.5
4	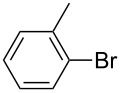	32.5	28.6	16.0
5	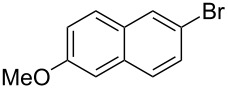	80.6	71.1	74.2

^a^General conditions: Ligand (2.5 × 10^−3^ mmol for HUGPHOS-1,2; 1.25 × 10^−3^ mmol for WIDEPHOS), [Pd(OAc)_2_] (2.5 × 10^−3^ mmol), aryl bromide (0.25 mmol), styrene (0.50 mmol), Cs_2_CO_3_ (0.50 mmol), DMF (0.750 mL), decane (0.025 mL), *T* = 130 °C, 1 h. ^b^Conversions were determined by GC using decane as internal standard.

**Scheme 9:**
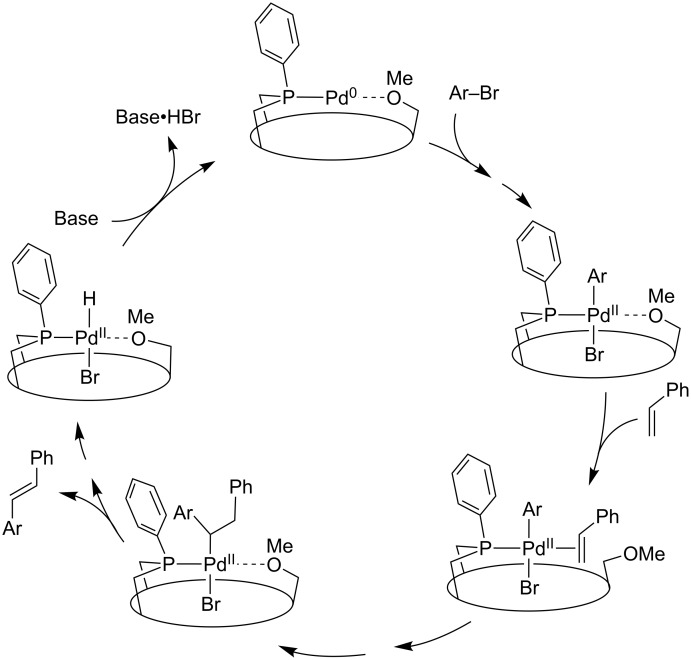
Simplified Heck coupling mechanism when using HUGPHOS-1 or HUGPHOS-2 as ligands. Doted lines stand for labile Pd–O bonds.

According to a number of mechanistic studies, the structure of the catalytic intermediates of Mizoroki–Heck reactions is strongly dependent on the phosphine used [73–81]. With very bulky monophosphines, active species having only one phosphine coordinated to the metal have been proposed [80,82]. In view of the above complexation studies, such mono-ligated intermediates are also likely to be operative with HUGPHOS ligands (Scheme 9). The fact that the observed reaction rates are higher with HUGPHOS ligands than with other bulky phosphines may be related to the presence of hemilabile methoxy groups [83] able either to stabilise highly reactive intermediates or assist the reduction–elimination step [80,82].

## Conclusion

In conclusion, we have shown that the cavity-shaped phosphines HUGPHOS-1 and HUGPHOS-2 exclusively form monophosphine complexes with Pd(II), Pt(II), Rh(I), and Ru(II) cations. In all these complexes, the CD cavity tightly embraces the metal centre. Such a feature has a strong influence on the catalytic outcome of both olefin hydroformylation and Heck coupling reactions. Despite being generally regarded as incompatible, both high regio- and high enantioselectivity were observed with HUGPHOS ligands in the Rh-catalysed hydroformylation of styrene. In these systems, the high isoselectivity arises from the ligand ability to exclusively generate monophosphine complexes while the high enantioselectivity is a result of the efficient chirality transfer imposed by the embracing character of the chiral CD cavity. To the best of our knowledge, the chiral inductions obtained with these ligands are the highest ever observed for a CD-derived catalyst operating in organic media [46,84–88]. Future work is aimed at extending the applications of HUGPHOS ligands to other metal-catalysed reactions.

## Experimental

All commercial reagents were used as supplied. All manipulations were performed in Schlenk-type flasks under N_2_. Solvents were dried by conventional methods and distilled immediately prior to use. Column chromatography was performed on silica gel 60 (particle size 40–63 μm, 230–240 mesh). CDCl_3_ was passed down a 5 cm thick alumina column and stored under N_2_ over molecular sieves (3 Å). Routine ^1^H, ^13^C{^1^H} and ^31^P{^1^H} NMR spectra were recorded with Bruker FT instruments (AVANCE 300, 400, 500, 600 spectrometers). ^1^H NMR spectral data were referenced to residual protiated solvents (δ = 7.26 ppm for CDCl_3_), ^13^C chemical shifts are reported relative to deuterated solvents (δ = 77.16 ppm for CDCl_3_) and the ^31^P NMR data are given relative to external H_3_PO_4_. Mass spectra were recorded either on a Maldi TOF spectrometer (MALDI–TOF) using α-cyano-4-hydroxycinnamic acid as matrix, or on a Bruker MicroTOF spectrometer (ESI–TOF) using CH_2_Cl_2_, MeCN or MeOH as the solvent. Elemental analyses were performed by the Service de Microanalyse, Institut de Chimie UMR 7177, Strasbourg. Melting points were determined with a Büchi 535 capillary melting point apparatus. The catalytic solutions containing the aldehydes were analysed by using a Varian 3900 gas chromatograph equipped with a WCOT fused-silica column (25 m × 0.25 mm). This allowed to determine the b:l ratio. In order to determine the enantiomeric excess, a sample of the reaction mixture (toluene) was treated with LiAlH_4_ for 0.5 h. After filtration, the toluene solution containing enantiomeric alcohols was analysed by GC with a Chirasil-DEX CB column (25 m × 0.25 mm). HUGPHOS-1, HUGPHOS-2 [38], WIDEPHOS [39], and [PdCl(dmba)]_2_ [89], were synthesized according to literature procedures. The synthesis and characterisation of compounds **5**, **9**, **12** and **13,** as well as the general procedure for rhodium catalysed hydroformylation reactions have been reported in a preliminary communication [44]. In the present full paper, the glucose units are ranged clockwise when looking at the primary face. The numbering of the atoms within a glucose unit is as follows:


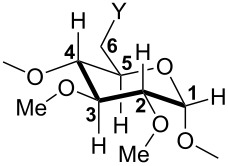


**2**: A solution of [(PdCl(dmba)]_2_ (0.170 g, 0.30 mmol) in CH_2_Cl_2_ (20 mL) was added to a solution of HUGPHOS-2 (0.900 g, 0.61 mmol) in CH_2_Cl_2_ (10 mL) under vigorous stirring at room temperature. After 15 min, the solvent was removed in vacuo and the residue was subjected to column chromatography (CH_2_Cl_2_/MeOH, 97:3, v/v) to afford pure **2** (0.900 g, 84%) as a pale yellow solid. *R*_f_ (SiO_2_, CH_2_Cl_2_/MeOH, 94:6, v/v) = 0.35; mp dec. >250 °C; ^1^H NMR (300.1 MHz, CDCl_3_, 25 °C) δ (assignment by COSY, ROESY and HMQC) 1.73–1.86 (m, 1H, H-6a^A^), 2.51–2.59 (m, 1H, H-6a^B^), 2.66 (s, 3H, NMe), 2.88 (s, 3H, NMe), 3.03–3.31 (10H, H-2, H-4^A,B^, H-6), 3.12 (s, 3H, OMe), 3.36 (s, 3H, OMe), 3.41 (s, 3H, OMe), 3.43 (s, 6H, OMe), 3.44 (s, 3H, OMe), 3.45 (s, 3H, OMe), 3.47 (s, 3H, OMe), 3.48 (s, 6H, OMe), 3.51 (s, 6H, OMe), 3.60 (s, 3H, OMe), 3.62 (s, 6H, OMe), 3.63 (s, 3H, OMe), 3.66 (s, 3H, OMe), 3.70, (s, 3H, OMe), 3.71 (s, 3H, OMe), 3.40–3.91 (29H, H-3, H-4, H-5, H-6, H-6b^A,B^, NCH_2_), 3.98 (d, 1H, ^2^*J*_H-6b,H-6a_ = 10.1 Hz, H-6), 4.17 (d, 1H, ^2^*J* = 12.9 Hz, NCH_2_), 4.49 (d, 1H, ^3^*J*_H-5,H-6_ = 9.3 Hz, H-5), 4.86 (d, 1H, ^3^*J*_H-1,H-2_ = 1.7 Hz, H-1), 4.87 (m, 1H, H-5^B^), 4.88 (d, 1H, ^3^*J*_H-1,H-2_ = 3.9 Hz, H-1), 5.00 (d, 1H, ^3^*J*_H-1,H-2_ = 3.3 Hz, H-1), 5.03 (d, 1H, ^3^*J*_H-1,H-2_ = 4.6 Hz, H-1), 5.04 (m, 1H, H-5^A^), 5.07 (d, 1H, ^3^*J*_H-1,H-2_ = 3.1 Hz, H-1), 5.09 (d, 1H, ^3^*J*_H-1,H-2_ = 4.0 Hz, H-1), 5.10 (d, 1H, ^3^*J*_H-1,H-2_ = 3.8 Hz, H-1), 6.31 (t, 1H, *J* = 6.9 Hz, *o*-H of dmba), 6.46 (t, 1H, *J* = 7.5 Hz, *m*-H of dmba), 6.78 (t, 1H, *J* = 7.3 Hz, *p*-H of dmba), 6.93 (d, 1H, *J* = 7.5 Hz, *m*-H of dmba), 7.23–7.31 (m, 3H, *m*-H, *p*-H), 7.65–7.71 (m, 2H, *o*-H) ppm; ^13^C{^1^H} NMR (75.5 MHz, CDCl_3_, 25 °C) δ (assignment by HMQC) 32.94 (d, ^1^*J*_C,P_ = 23.5 Hz, C-6^A^), 33.6 (d, ^1^*J*_C,P_ = 29.4 Hz, C-6^B^), 49.63 (br s, NCH_3_), 50.44 (br s, NCH_3_), 57.81, 57.96, 58.04, 58.19 [×2], 58.65, 58.75, 58.85 [×2], 58.90 [×2], 59.09, 61.21, 61.30, 61.56, 61.62, 61.70 [×2], 62.05 (OMe), 66.25 (d, ^2^*J*_C,P_ = 5.8 Hz, C-5^A^), 70.17 (d, ^2^*J*_C,P_ = 7.8 Hz, C-5^B^), 70.90 [×2], 71.09 [×2], 71.75 (C-5), 71.09, 71.32, 71.52, 71.64, 72.36 (C-6), 73.01 (NCH_2_), 79.91, 79.98, 80.58, 80.89 [×2], 80.98, 81.19, 81.74 [×3], 82.17, 82.36, 82.53, 82.68 [×2], 82.82, 82.94, 83.08, 83.45 (C-2, C-3, C-4), 88.02 (d, ^3^*J*_C,P_ = 9.5 Hz, C-4^A^), 88.65 (d, ^3^*J*_C,P_ = 4.4 Hz, C-4^B^), 96.57, 97.94, 98.79, 100.20 [×2], 100.29, 101.58 (C-1), 122.01 (d, ^4^*J*_C,P_ = 2.3 Hz, dmba-C*_meta_*), 123.52 (dmba-C*_para_*), 125.36 (d, ^4^*J*_C,P_ = 6.0 Hz, dmba-C*_meta_*), 128.15 (d, ^3^*J*_C,P_ = 10.4 Hz, dmba-C*_ortho_*_-H_), 130.15 (C*_para_*), 132.38 (d, ^2^*J*_C,P_ = 10.8 Hz, C*_ortho_*), 134.91 (d, ^1^*J*_C,P_ = 49.0 Hz, C*_ipso_*), 136.47 (d, ^3^*J*_C,P_ = 10.0 Hz, C*_meta_*), 147.67 (dmba-C*_ortho_*_-C_), 152.44 (dmba-C*_ipso_*) ppm; ^31^P{^1^H} NMR (121.5 MHz CDCl_3_, 25 °C) δ 25.5 (s) ppm; elemental analysis (%) calcd for C_76_H_123_ClNO_33_PPd (1751.62): C, 52.11; H, 7.08; N, 0.80; found: C, 52.34; H, 7.07;, N, 0.80; MS (ESI–TOF): *m*/*z* (%): 1714.67 (100) [M – Cl]^+^, 1774.64 (4) [M + Na]^+^, 1790.60 (8) [M + K]^+^.

**6**: Water was removed over 12 h by azeotropic distillation of a toluene solution (100 mL) of compound **5** (0.100 g, 0.06 mmol) using a Dean–Stark apparatus. After allowing the solution to reach room temperature, the solvent was removed in vacuo affording quantitatively compound **6** (0.098 g, 0.06 mmol) as a colourless solid. mp dec. >250 °C; ^1^H NMR (300.1 MHz, CDCl_3_, 25 °C) δ (assignment by COSY and HMQC) 2.37 (m, 1H, H-6a^A^), 2.59 (m, 1H, H-6a^B^), 2.69 (dd, 1H, ^3^*J*_H-2,H-3_ = 9.9 Hz, ^3^*J*_H-2,H-1_ = 2.8 Hz, H-2), 2.93–3.07 (2H, H-6b^A,B^), 3.12–3.26 (5H, H-2), 3.34 (m, 3H, OMe), 3.38 (s, 3H, OMe), 3.39 (s, 3H, OMe), 3.40 (s, 3H, OMe), 3.43 (s, 3H, OMe), 3.48 (s, 6H, OMe), 3.51 (s, 12H, OMe), 3.56 (m, 3H, OMe), 3.60 (s, 3H, OMe), 3.61 (s, 3H, OMe), 3.63 (s, 6H, OMe), 3.64 (s, 3H, OMe), 3.65 (s, 3H, OMe), 3.68 (m, 3H, OMe), 3.30–3.93 (27H, H-2, H-3, H-4, H-5, H-6), 3.99 (dd, 1H, ^2^*J*_H-6b,H-6a_ = 9.7 Hz, ^3^*J*_H-6b,H-5_ = 2.7 Hz, H-6), 4.19 (dd, 1H, ^2^*J*_H-6b,H-6a_ = 11.0 Hz, ^3^*J*_H-6b,H-5_ = 2.4 Hz, H-6), 4.25 (m, 1H, H-5), 4.42 (m, 1H, H-5^A^), 4.50 (m, 1H, H-5^B^), 4.85 (d, 1H, ^3^*J*_H-1,H-2_ = 4.7 Hz, H-1), 4.98 (d, 1H, ^3^*J*_H-1,H-2_ = 2.6 Hz, H-1), 5.06 (d, 1H, ^3^*J*_H-1,H-2_ = 3.7 Hz, H-1), 5.08 (d, 1H, ^3^*J*_H-1,H-2_ = 3.6 Hz, H-1), 5.11 (d, 1H, ^3^*J*_H-1,H-2_ = 3.1 Hz, H-1), 5.15 (d, 1H, ^3^*J*_H-1,H-2_ = 3.1 Hz, H-1), 5.21 (d, 1H, ^3^*J*_H-1,H-2_ = 3.1 Hz, H-1), 7.33–7.45 (3H, *m*-H, *p*-H), 7.97 (ddd, 2H, ^3^*J_o_*_-H,P_ = 12.1 Hz, ^3^*J_o_*_-H,_*_m_*_-H_ = 6.9 Hz, ^3^*J_o_*_-H,_*_p_*_-H_ = 1.0 Hz, *o*-H) ppm; ^13^C{^1^H} NMR (75.5 MHz, CDCl_3_, 25 °C) δ (assignment by HMQC) 27.94–28.09 (m, C-6^A,B^), 57.89, 58.29 [×2], 58.59 [×2], 58.62 [×2], 59.01, 59.05, 59.11, 59.25 [×2], 60.77, 60.98, 61.04, 61.32, 61.36, 61.65, 61.71 (OMe), 65.36 (d, ^2^*J*_C,P_ = 8.6 Hz, C-5^B^), 69.30 (d, ^2^*J*_C,P_ = 3.9 Hz, C-5^A^), 70.80, 71.09 [×2], 71.26, 71.52 (C-5), 71.10, 71.36 [×2], 71.43, 71.61 (C-6), 79.15, 79.39, 79.91, 80.03, 80.12, 80.82, 80.87, 81.19, 81.32, 81.42, 81.49, 81.84 [×2], 81.89 [×2], 82.10, 82.24, 82.35, 82.41, 83.53, 84.17 (C-2, C-3, C-4), 96.98, 97.26, 98.79, 98.97, 99.36, 99.47, 99.64 (C-1), 128.57 (C*_para_*), 128.97 (d, ^3^*J*_C,P_ = 5.8 Hz, C*_meta_*), 131.46 (d, ^1^*J*_C,P_ = 50.6 Hz, C*_ipso_*), 132.53 (d, ^2^*J*_C,P_ = 10.5 Hz, C*_ortho_*) ppm; ^31^P{^1^H} NMR (121.5 MHz CDCl_3_, 25 °C) δ 19.8 (s) ppm; MS (ESI–TOF): *m*/*z* (%): 1673.52 (40) [M + Na]^+^. We do not provide microanalytical data for this compound because of fast rehydration in air.

**7** and **8**: A solution of HUGPHOS-2 (0.126 g, 0.09 mmol) in CH_2_Cl_2_ (5 mL) was added dropwise to a solution of [RuCl_2_(η^6^-*p*-cymene)]_2_ (0.052 g, 0.08 mmol) in CH_2_Cl_2_ (5 mL) under vigorous stirring at room temperature. The solution was stirred for 1 h before being evaporated to dryness. The crude product was purified by column chromatography (SiO_2_, CH_2_Cl_2_/MeOH, 97:3 to 95:5, *v*/*v*) affording **7** (0.085 g, 56%) and **8** (0.065 g, 42%) as brown solids. **7**: mp 210 °C; ^1^H NMR (500.1 MHz, CDCl_3_, 25 °C) δ (assignment by combined COSY, ROESY and HSQC) 1.13 (d, 3H, ^3^*J*_CH3,CH_ = 7.1 Hz, CH_3_^iPr^ of *p*-cymene), 1.15 (d, 3H, ^3^*J*_CH3,CH_ = 7.1 Hz, CH_3_^iPr’^ of *p*-cymene), 1.63 (s, 3H, CH_3_ of *p*-cymene), 2.57 (ddd, 1H, ^2^*J*_H-6a,H-6b_ = 15.3 Hz, ^2^*J*_H-6a,P_ = 11.4 Hz, ^3^*J*_H-6a,H-5_ = 2.5 Hz, H-6a^A^), 2.71 (sept, 1H, ^3^*J*_CH,CH3_ = 7.1 Hz, CH^iPr^ of *p*-cymene), 3.00 (dd, 1H, ^3^*J*_H-2,H-1_ = 2.9 Hz, ^3^*J*_H-2,H-3_ = 9.8 Hz, H-2), 3.19 (s, 3H, OMe), 3.34 (s, 3H, OMe), 3.35 (s, 3 H, OMe), 3.40 (s, 3H, OMe), 3.41 (s, 3H, OMe), 3.45 (s, 3H, OMe), 3.46 (s, 3H, OMe), 3.47 (s, 3H, OMe), 3.48 (s, 3H, OMe), 3.51 (s, 3H, OMe), 3.52 (s, 3H, OMe), 3.53 (s, 3H, OMe), 3.54 (s, 3H, OMe), 3.56 (s, 3H, OMe), 3.58 (s, 3H, OMe), 3.62 (s, 3H, OMe), 3.63 (s, 3H, OMe), 3.69 (s, 3H, OMe), 3.82 (s, 3H, OMe), 3.06–3.85 (36H, H-2, H-3, H-4, H-5, H-6), 3.88 (dd, 1H, ^2^*J*_H-6a,H-6b_ = 10.8 Hz, ^3^*J*_H-6a,H-5_ = 4.3 Hz, H-6), 3.91–3.95 (m, 1H, H-5), 4.02–4.10 (2 H, H-5, H-5^A^), 4.82 (d, 1H, ^3^*J*_H-1,H-2_ = 2.8 Hz, H-1), 4.88 (dd, 1H, ^3^*J_m_*_-H,_*_o_*_-H_ = 5.6 Hz, ^3^*J_m_*_-H,_*_m_*_-H’_ = 2.3 Hz, *m*-H of *p*-cymene), 4.95 (d, 1H, ^3^*J*_H-1,H-2_ = 4.1 Hz, H-1), 5.04 (d, 1H, ^3^*J*_H-1,H-2_ = 3.9 Hz, H-1), 5.05 (d, 1H, ^3^*J*_H-1,H-2_ = 3.5 Hz, H-1), 5.06–5.09 (2H, H-1, *o*-H of *p*-cymene), 5.14–5.18 (2H, H-1, *o*-H’ of *p*-cymene), 5.26–5.29 (m, 1H, *m*-H’ of *p*-cymene), 5.36 (d, 1H, ^3^*J*_H-1,H-2_ = 4.1 Hz, H-1), 7.45–7.56 (3H, *m*-H, *p*-H), 7.91–7.96 (2 H, *o*-H) ppm; ^13^C{^1^H} NMR (125.8 MHz, CDCl_3_, 25 °C) δ (assignment by HSQC) = 16.32 (CH_3_ of *p*-cymene), 21.04, 21.10 (CH_3_^iPr^ of *p*-cymene), 27.26 (d, ^1^*J*_C,P_ = 27.8 Hz, C-6^A^), 27.36 (d, ^1^*J*_C,P_ = 23.8 Hz, C-6^B^), 29.50 (CH of *p*-cymene), 55.99, 56.99, 57.61, 57.74, 57.77, 57.83, 58.05, 58.13, 58.16, 58.26, 58.33, 59.00, 59.69, 60.01, 60.24, 60.44, 60.61, 60.64, 60.81, 63.79 (d, ^2^*J*_C,P_ = 9.4 Hz, C-5^A^), 68.48 (d, ^2^*J*_C,P_ = 10.1 Hz, C-5^B^), 69.88 [×2] (C-6), 69.94 [×2], 70.39 (C-5), 70.46, 70.55 (C-6), 70.70 (C-5), 70.82 (C-6), 71.76 (C-5), 76.84, 78.21, 79.16, 79.80 [×2], 79.93, 80.09, 80.34 [×2], 80.45, 80.61, 80.68, 81.01, 81.20, 81.32 [×2], 81.61 [×2], 81.93, 82.74, 84.46, 87.88 (C-2, C-3, C-4, C*_ortho_* of *p*-cymene), 88.64, 90.43 (C*_meta_* of *p*-cymene), 91.55 (C*_ipso_* of *p*-cymene), 95.27, 96.29, 96.73, 98.54, 98.74, 98.92 [×2] (C-1), 109.23 (C*_ipso_* of *p*-cymene), 127.74, 127.81 (C*_meta_*), 129.17 (C*_para_*), 130.30, 130.35 (C*_ortho_*), 132.65 (d, ^1^*J*_C,P_ = 38.3 Hz, C*_ipso_*) ppm; ^31^P{^1^H} NMR (161.9 MHz, CDCl_3_, 25 °C) δ 20.4 (s) ppm; elemental analysis (%) calcd for C_77_H_125_Cl_2_O_33_PRu·CH_2_Cl_2_ (1781.75 + 84.93): C, 50.19; H, 6.86; found: C, 50.05; H, 6.80; MS (ESI-TOF): *m/z* (%): 1803.62 (100) [*M* + Na]^+^. **8**: mp 210 °C; ^1^H NMR (500.1 MHz, CDCl_3_, 25 °C) δ (assignment by combined COSY, ROESY and HSQC) 0.94 (d, 3H, ^3^*J*_CH3,CH_ = 7.1 Hz, CH_3_^iPr^ of *p*-cymene), 1.05 (d, 3H, ^3^*J*_CH3,CH_ = 7.1 Hz, CH_3_^iPr’^ of *p*-cymene), 1.73 (s, 3H, CH_3_ of *p*-cymene), 2.28 (sept, 1H, ^3^*J*_CH,CH3_ = 7.1 Hz, CH^iPr^ of *p*-cymene), 2.50 (dt, 1H, ^2^*J*_H-6a,H-6b_ = 18.2 Hz, ^2^*J*_H-6a,P_ = ^3^*J*_H-6a,H-5_ = 7.2 Hz, H-6a^A^), 2.59 (dd, 1H, ^3^*J*_H-2,H-1_ = 3.1 Hz, ^3^*J*_H-2,H-3_ = 9.9 Hz, H-2^A^), 2.75 (ddd, 1H, ^2^*J*_H-6a,H-6b_ = 14.3 Hz, ^2^*J*_H-6a,P_ = 12.0 Hz, ^3^*J*_H-6a,H-5_ = 2.6 Hz, H-6a^B^), 2.88 (t, 1H, ^3^*J*_H-4,H-3_ = ^3^*J*_H-4,H-5_ = 9.5 Hz, H-4^A^), 3.12–3.27 (7H, H-2, H-6b^B^), 3.35 (s, 3H, OMe), 3.36 (s, 3H, OMe), 3.38 (s, 3H, OMe), 3.39 (s, 3H, OMe), 3.40 (s, 3H, OMe), 3.41 (s, 3H, OMe), 3.45 (s, 3H, OMe), 3.46 (s, 3H, OMe), 3.48 (s, 6H, OMe), 3.49 (s, 3H, OMe), 3.53 (s, 3H, OMe), 3.56 (s, 3H, OMe), 3.57 (s, 3H, OMe), 3.58 (s, 3H, OMe), 3.61 (s, 3H, OMe), 3.62 (s, 3H, OMe), 3.63 (s, 3H, OMe), 3.64 (s, 3H, OMe), 3.33–3.87 (26H, H-3, H-4, H-5, H-6), 3.92 (dd, 1H, ^2^*J*_H-6a,H-6b_ = 10.3 Hz, ^3^*J*_H-6a,H-5_ = 2.5 Hz, H-6), 4.10 (d, 1H, ^2^*J*_H-6a,H-6b_ = 10.3 Hz, H-6), 4.21–4.32 (2H, H-5^B^, H-6), 4.35 (ddd, 1H, ^2^*J*_H-5,H-6a_ = 7.2 Hz, ^2^*J*_H-5,H-6b_ = 26.1 Hz, ^3^*J*_H-5,H-4_ = 10.9 Hz, H-5^A^), 4.73 (d, 1H, ^3^*J*_H-1,H-2_ = 5.1 Hz, H-1), 4.74 (d, 1H, ^3^*J_o_*_-H,_*_m_*_-H_ = 5.9 Hz, *o*-H’ of *p*-cymene), 4.95 (d, 1H, ^3^*J_m_*_-H,_*_o_*_-H_ = 5.9 Hz, *m*-H’ of *p*-cymene), 4.96 (d, 1H, ^3^*J*_H-1,H-2_ = 2.4 Hz, H-1), 4.98 (d, 1H, ^3^*J*_H-1,H-2_ = 2.7 Hz, H-1), 5.06 (d, 1H, ^3^*J*_H-1,H-2_ = 3.1 Hz, H-1), 5.10 (d, 1H, ^3^*J*_H-1,H-2_ = 3.2 Hz, H-1), 5.13 (d, 1H, ^3^*J_m_*_-H,_*_o_*_-H_ = 6.5 Hz, *m*-H of *p*-cymene), 5.15 (d, 1H, ^3^*J*_H-1,H-2_ = 3.4 Hz, H-1), 5.16 (d, 1H, ^3^*J*_H-1,H-2_ = 3.4 Hz, H-1), 5.25 (d, 1H, ^3^*J_o_*_-H,_*_m_*_-H_ = 6.5 Hz, *o*-H of *p*-cymene), 7.32–7.40 (3H, *m*-H, *p*-H), 8.03–8.11 (2H, *o*-H) ppm; ^13^C{^1^H} NMR (125.8 MHz, CDCl_3_, 25 °C) δ (assignment by HSQC) 16.77 (CH_3_ of *p*-cymene), 20.80, 21.24 (CH_3_^iPr^ of *p*-cymene), 23.46 (d, ^1^*J*_C,P_ = 25.8 Hz, C-6^A^), 28.57 (d, ^1^*J*_C,P_ = 26.0 Hz, C-6^B^), 28.95 (CH of *p*-cymene), 56.36, 56.97, 57.09, 57.63, 57.68, 57.83 [×2], 57.99, 58.03, 58.12, 58.19, 58.61, 59.40, 60.19, 60.31, 60.42, 60.46, 60.93, 60.97 (OMe), 65.82 (d, ^2^*J*_C,P_ = 11.1 Hz, C-5^B^), 67.61 (d, ^2^*J*_C,P_ = 9.3 Hz, C-5^A^), 69.61 (C-5), 69.63 (C-6), 69.75, 69.93 (C-5), 69.99, 70.06 [×2], 70.13 (C-6), 70.34, 70.96 (C-5), 74.88 (d, ^3^*J*_C,P_ = 4.4 Hz, C-4^A^), 79.29, 79.34, 79.40, 79.55, 80.06 [×2], 80.48, 80.53, 80.62 [×2], 80.67, 80.69, 80.81, 80.85, 80.95, 81.23, 81.26, 81.41, 82.89 (C-2, C-3, C-4), 83.45, 83.65 (C*_ortho_* of *p*-cymene), 83.84 (C*_meta_* of *p*-cymene), 84.43 (C-4^B^), 88.01 (C*_meta_* of *p*-cymene), 95.54 (C-1), 95.62 (C*_ipso_* of *p*-cymene), 96.42, 97.94, 98.35, 98.76 [×2], 98.86 (C-1), 108.11 (C*_ipso_* of *p*-cymene), 126.06, 126.13 (C*_meta_*), 128.94 (C*_para_*), 130.68, 130.82 (C*_ortho_*), 134.91 (d, ^1^*J*_C,P_ = 38.5 Hz, C*_ipso_*) ppm; ^31^P{^1^H} NMR (161.9 MHz, CDCl_3_, 25ºC) δ 22.8 (s) ppm; elemental analysis (%) calcd for C_77_H_125_Cl_2_O_33_PRu·CH_2_Cl_2_ (1781.75 + 84.93): C, 50.19; H, 6.86; found: C, 50.03; H, 6.78; MS (ESI–TOF): *m*/*z* (%): 1803.62 (100) [M + Na]^+^.

**10a** and **10b**: A solution of HUGPHOS-2 (0.100 g, 0.07 mmol) in CH_2_Cl_2_ (5 mL) was added dropwise to a solution of [Rh(CO)_2_Cl]_2_ (0.016 g, 0.04 mmol) in CH_2_Cl_2_ (5 mL) under vigorous stirring at room temperature. The reaction mixture was stirred for 1 h before being evaporated to dryness in vacuo to afford quantitatively a mixture of **10a** and **10b** (**10a/10b**, 85:15, 0.114 g, 99%) as a brown solid. *R*_f_ (SiO_2_) = dec; mp > 250 °C; Selected spectroscopic data: ^13^C{^1^H} NMR (125.8 MHz, CDCl_3_, 25 °C) δ 181.12–182.82 (m, CO), 187.24–189.73 (m, CO) ppm; ^31^P{^1^H} NMR (161.9 MHz, CDCl_3_, 25 °C) δ 13.9 (**10b,** d, ^1^*J*_P,Rh_ = 121 Hz), 20.1 (**10a,** d, ^1^*J*_P,Rh_ = 124 Hz) ppm; IR: 2090 (s, CO), 2005 (s, CO), 1985 (s, CO) cm^−1^; elemental analysis (%) calcd for C_69_H_111_ClO_35_PRh·3CH_2_Cl_2_ (1669.93 + 254.80): C 44.99, H 6.31 found: C 45.01, H 6.99; MS (ESI–TOF): *m*/*z* (%): 1605.58 (100) [M – CO – Cl]^+^, 1663.54 (20) [*M* – CO + Na]^+^; MS (ESI–TOF): *m*/*z* (%): 1675.55 (100) [M – CO + Cl]^−^; MS (MALDI–TOF): *m*/*z* (%): 1605.58 (100) [M – CO – Cl]^+^, 1719.69 (5) [M + CO + Na]^+^.

**11**: A solution of [Rh(CO)_2_Cl]_2_ (0.080 g, 0.20 mmol) in CH_2_Cl_2_ (7 mL) was added dropwise to a solution of HUGPHOS-2 (0.100 g, 0.07 mmol) in CH_2_Cl_2_ (5 mL) under vigorous stirring at room temperature. After 1 h, the volume of the reaction mixture was reduced to 5 mL and pentane (40 mL) was added in order to precipitate unreacted [Rh(CO)_2_Cl]_2_, which was removed by filtration on a pad of Celite. The resulting solution was evaporated to dryness in vacuo to afford quantitatively **11** as a brown powder (0.103 g, 83%). mp dec >250 °C; ^1^H NMR (500.1 MHz, CDCl_3_, 25 °C) δ (assignment by combined COSY and HSQC) = 1.91 (q, 1H, ^2^*J*_H-6a,H-6b_ = ^2^*J*_H-6a,P_ = ^3^*J*_H-6a,H-5_ = 15.4 Hz, H-6a^B^), 2.17 (t, 1H, ^2^*J*_H-6a,H-6b_ = ^2^*J*_H-6a,P_ = 14.2 Hz, H-6a^A^), 3.02 (m, 1H, H-6b^B^), 3.20 (s, 3H, OMe), 3.30 (s, 3H, OMe), 3.32 (s, 3H, OMe), 3.36 (s, 3H, OMe), 3.45 (s, 6H, OMe), 3.46 (s, 3H, OMe), 3.48 (s, 3H, OMe), 3.50 (s, 6H, OMe), 3.52 (s, 3H, OMe), 3.53 (s, 3H, OMe), 3.59 (s, 6H, OMe), 3.62 (s, 3H, OMe), 3.64 (s, 3H, OMe), 3.65 (s, 3H, OMe), 3.68 (s, 3H, OMe), 3.76 (s, 3H, OMe), 3.13–3.79 (26H, H-2, H-3, H-4, H-6), 3.80–3.92 (4H, H-6), 3.93–4.01 (4H, H-5, H-6), 4.07–4.13 (3H, H-5, H-6), 4.36 (dt, 1H, ^3^*J*_H-5,H-4_ = ^3^*J*_H-5,H-6b_ = 10.5 Hz, ^3^*J*_H-5,H-6a_ = 15.4 Hz, H-5^B^), 4.95 (d, 1H, ^3^*J*_H-1,H-2_ = 3.7 Hz, H-1), 5.00 (d, 1H, ^3^*J*_H-1,H-2_ = 4.6 Hz, H-1), 5.03 (d, 1H, ^3^*J*_H-1,H-2_ = 3.7 Hz, H-1), 5.08–5.11 (3H, H-1), 5.20 (d, 1H, ^3^*J*_H-1,H-2_ = 3.7 Hz, H-1), 5.35–5.45 (m, 1H, H-5^A^), 7.42–7.46 (3H, *m*-H, *p*-H), 7.82–7.87 (2H, *o*-H) ppm; ^13^C{^1^H} NMR (125.8 MHz, CDCl_3_, 25 °C) δ (assignment by HSQC) = 32.32 (d, ^1^*J*_C,P_ = 29.3 Hz, C-6^A^), 35.77 (d, ^1^*J*_C,P_ = 29.3 Hz, C-6^B^), 56.74, 57.18, 57.26, 57.63 [×2], 57.78, 57.91, 57.96, 57.99, 58.06, 58.08, 58.29, 60.13, 60.31, 60.54 [×2], 60.56, 60.70, 60.83 (OMe), 63.30 (C-5^A^), 69.43, 69.50, 69.68, 69.90, 69.94 (C-5), 70.01, 70.24, 70.44, 70.60, 70.76 (C-6), 71.31 (d, ^2^*J*_C,P_ = 15.9 Hz, C-5^B^), 78.57, 79.04, 79.79 [×2], 80.17, 80.23 [×2], 80.47, 80.51, 80.57, 80.90, 81.05, 81.12, 81.32, 81.40, 81.68, 81.76, 82.14, 83.09 (C-2, C-3, C-4), 84.17 (d, ^3^*J*_C,P_ = 10.7 Hz, C-4^B^), 88.02 (d, ^3^*J*_C,P_ = 4.6 Hz, C-4^A^), 96.39, 97.66, 97.70, 98.52, 98.66, 99.37, 99.79 (C-1), 127.62, 127.70 (C*_meta_*), 130.08 (C*_para_*), 130.56, 130.64 (C*_ortho_*), 135.13 (d, ^1^*J*_C,P_ = 57.5 Hz, C*_ipso_*), 176.16 [×2] (d, ^1^*J*_C,Rh_ = 77.2 Hz, CO), 177.63 (dd, ^1^*J*_C,Rh_ = 71.9 Hz, ^2^*J*_C,P_ = 22.8 Hz, CO) ppm; ^31^P{^1^H} NMR (161.9 MHz, CDCl_3_, 25 °C) δ 40.5 (d, ^1^*J*_P,Rh_ = 172 Hz) ppm; IR: 2086 (vs, CO), 2026 (vs, CO), 2004 (s, CO) cm^−1^; We do not provide microanalytical data for this compound because of strong hydration; MS (ESI–TOF): *m*/*z* (%): 1799.44 (70) [M – Cl]^+^, 1857.40 (100) [M + Na]^+^.

**General procedure for palladium-catalysed Heck cross-coupling reactions**: In an oven-dried Schlenk tube, a solution of [Pd(OAc)_2_] in DMF, a solution of HUGPHOS-1/2 or WIDEPHOS ligands in DMF, aryl bromide (1 equiv), styrene (2 equiv), Cs_2_CO_3_ (2 equiv), decane (internal reference) and additional DMF were introduced under an inert atmosphere. The reaction mixture was heated for 1 h. After cooling to room temperature, a small amount (0.5 mL) of the resulting solution was passed through a Millipore filter and analyzed by GC. Each experiment was repeated twice and the conversion values given in Table 3 and Table 4 correspond to the mean value from two catalytic tests.

## Supporting Information

File 1 Copies of NMR spectra for compounds **2**, **3**, **6**, **7**, **8**, **10a** and **10b** and **11**.
