# Supplementary material for: Phosphinocyclodextrins as confining units for catalytic metal centres. Applications to carbon–carbon bond forming reactions
Source: Beilstein J Org Chem. 2014 Oct 15;10:2388–405. doi: 10.3762/bjoc.10.249 (PMC4222288; doi:10.3762/bjoc.10.249)
Supplement: File 1 — Copies of NMR spectra for compounds 2, 3, 6, 7, 8, 10a and 10b and 11. [file Beilstein_J_Org_Chem-10-2388-s001.pdf]

Supporting Information

for

Phosphinocyclodextrins as confining units for catalytic metal centres. Applications to carbon–carbon bond forming reactions

Matthieu Jouffroy[1], Rafael Gramage-Doria[1], David Sémeril[1], Dominique Armspach*[1], Dominique Matt*[1], Werner Oberhauser[2] and Loïc Toupet[3]

Address: [1]Laboratoire de Chimie Inorganique Moléculaire et Catalyse, Institut de Chimie UMR 7177 CNRS, Université de Strasbourg, 1, rue Blaise Pascal, F-67008 Strasbourg Cedex, France, [2]Istituto di Chimica dei Composti OrganoMetallici CNR, via Madonna del Piano, 10, 50019 Sesto Fiorentino, Firenze, Italy, [3]Dr. L. Toupet, Groupe Matière Condensée et Matériaux, UMR 6626 CNRS, Université de Rennes 1, 263, avenue du Général Leclerc, 35042 Rennes Cedex, France

Email: Dominique Armspach* - d.armspach@unistra.fr; Dominique Matt* - dmatt@unistra.fr

*Corresponding author

Copies of NMR spectra for compounds 2, 3, 6, 7, 8, 10a and 10b and 11.

Formula of compounds 2, 3, 6-8, 10a,b, and 11

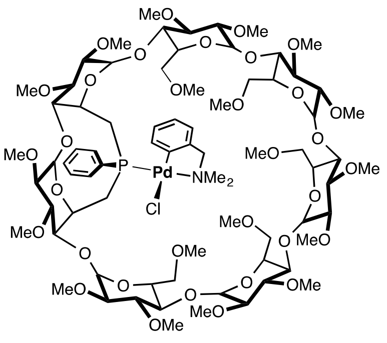

2

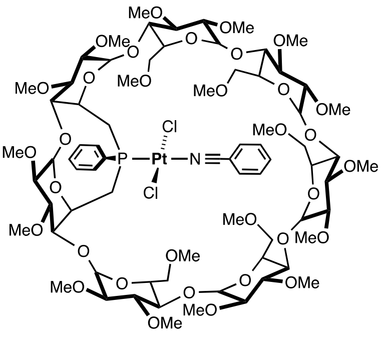

3

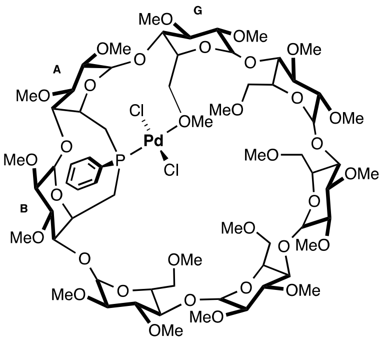

6

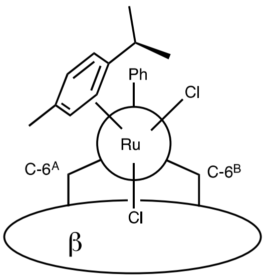

7

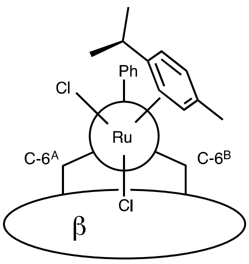

8

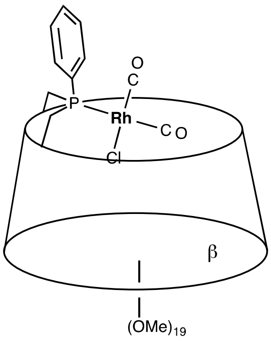

10a

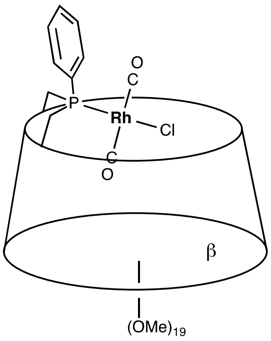

10b

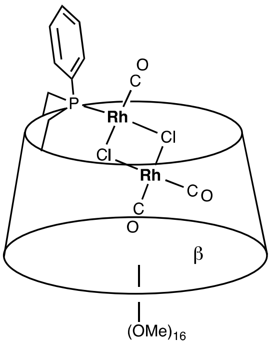

11

Copies of NMR spectra for compounds 2, 3, 6, 7, 8, 10a and 10b and 11.

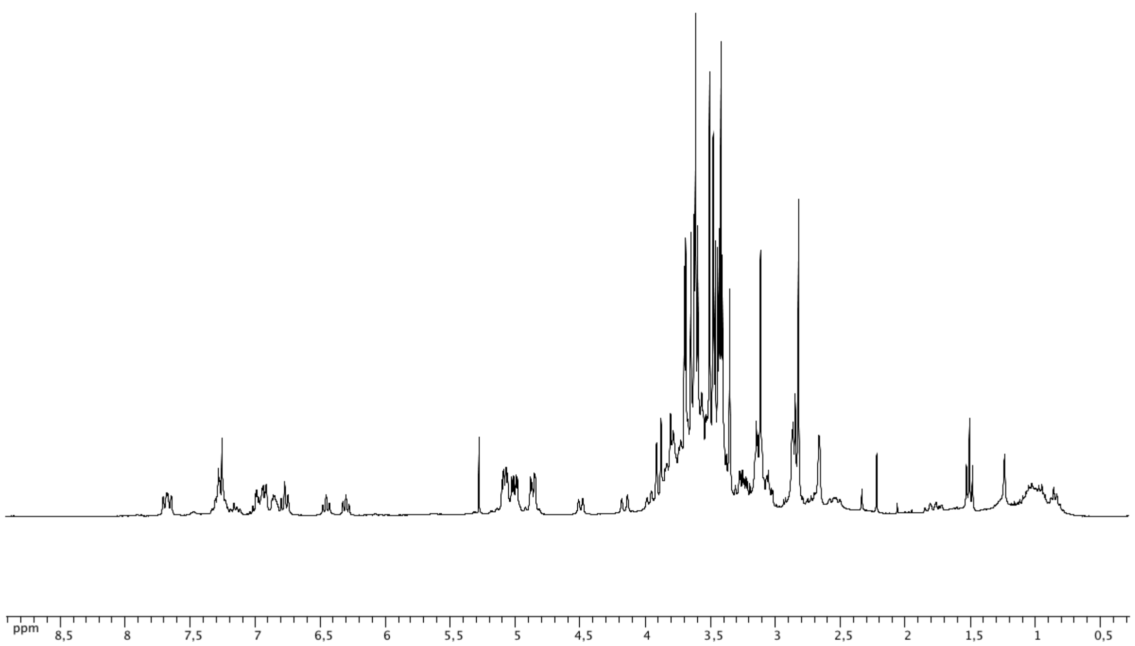

^{1}H NMR spectrum of **2** (400 MHz, $CDCl_3$, 25 °C)

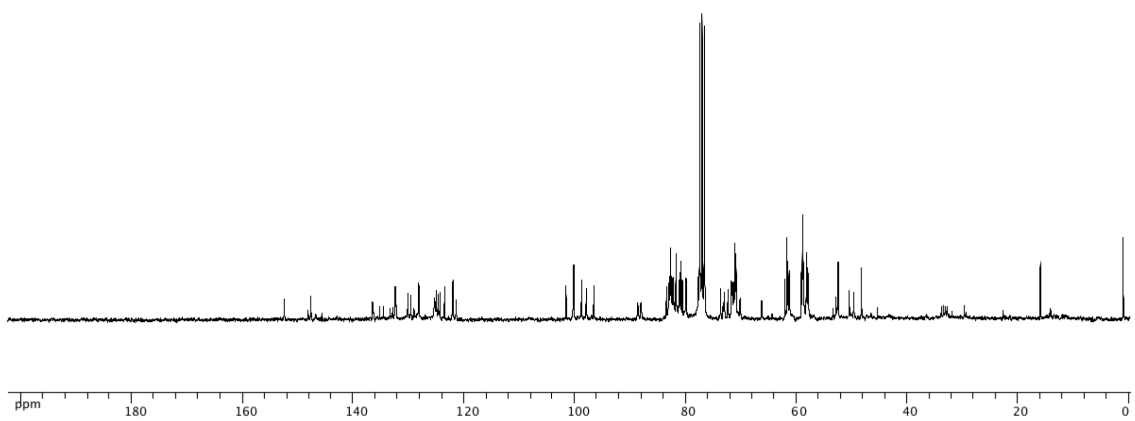

^{13}C{^{1}H} NMR spectrum of **2** (125 MHz, $CDCl_3$, 25 °C)

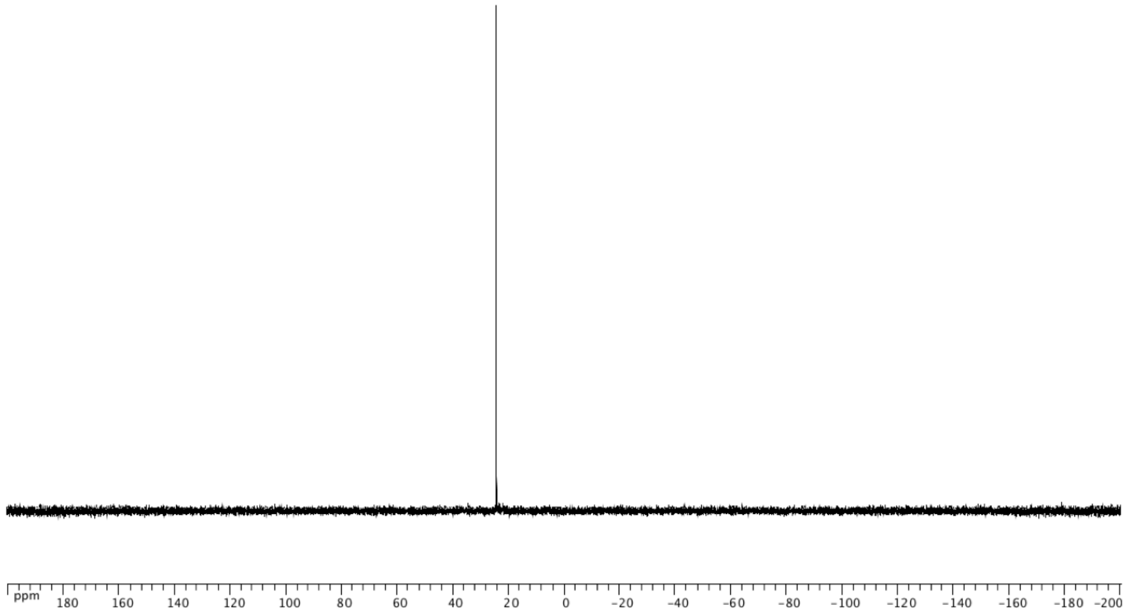

$^{31}P\{^{1}H\}$ NMR spectrum of **2** (160 MHz, $CDCl_3$, 25 °C)

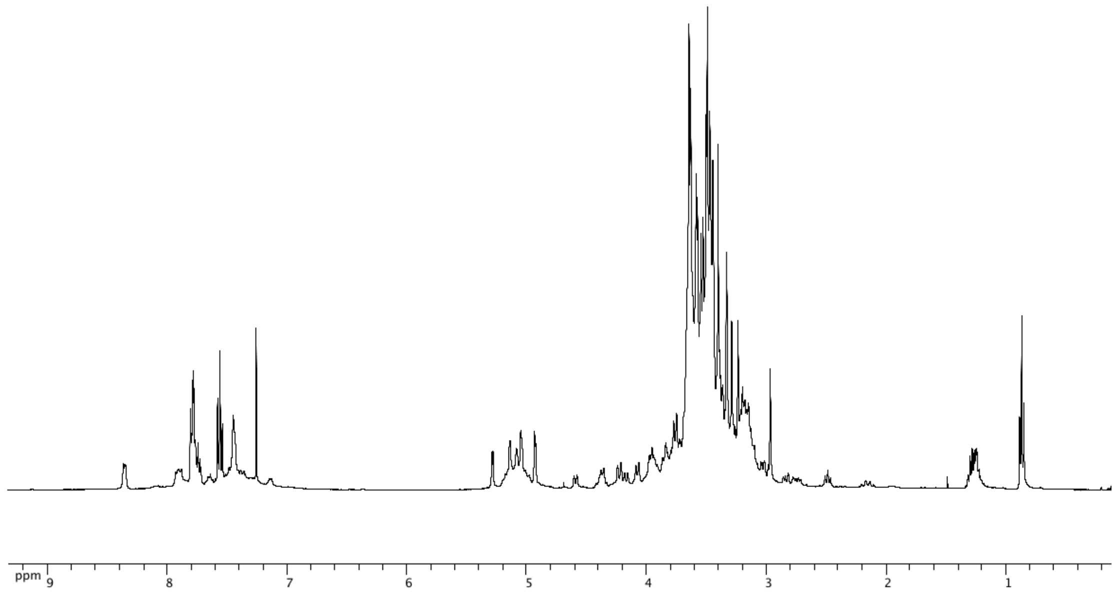

^{1}H NMR spectrum of **3** (400 MHz, $CDCl_3$, 25 °C)

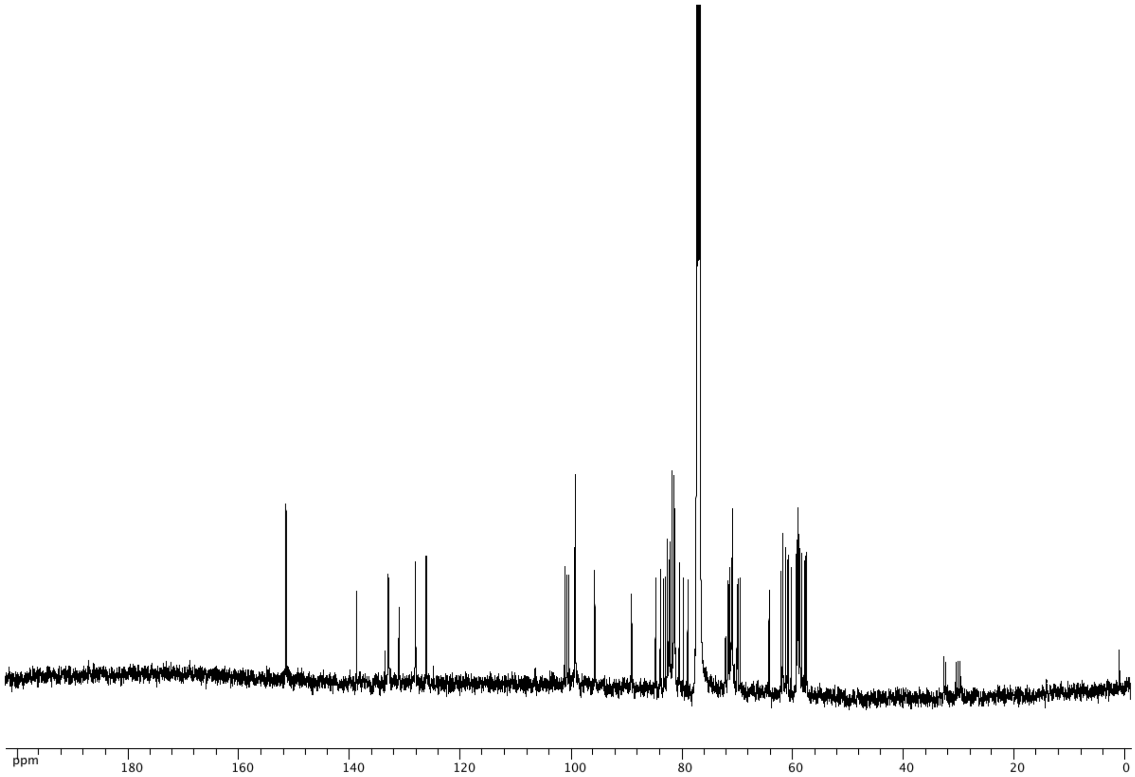

^{13}C{^{1}H} NMR spectrum of **3** (125 MHz, $CDCl_3$, 25 °C)

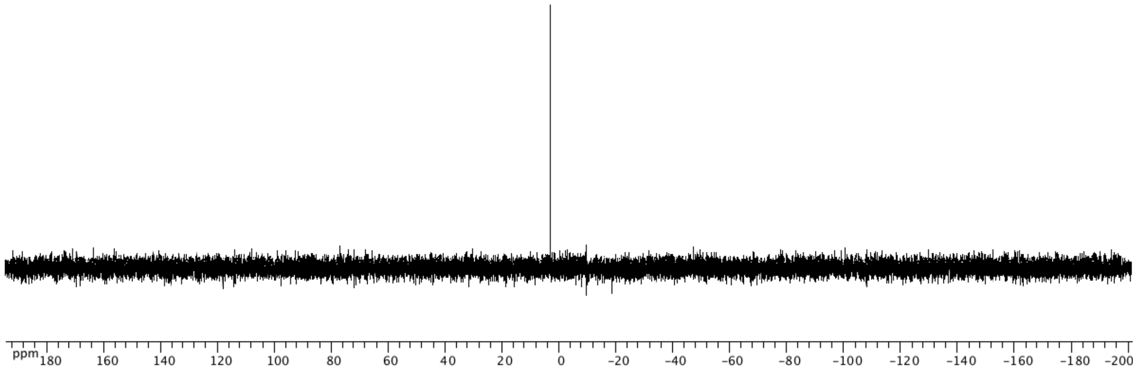

$^{31}P\{^{1}H\}$ NMR spectrum of **3** (160 MHz, $CDCl_3$, 25 °C)

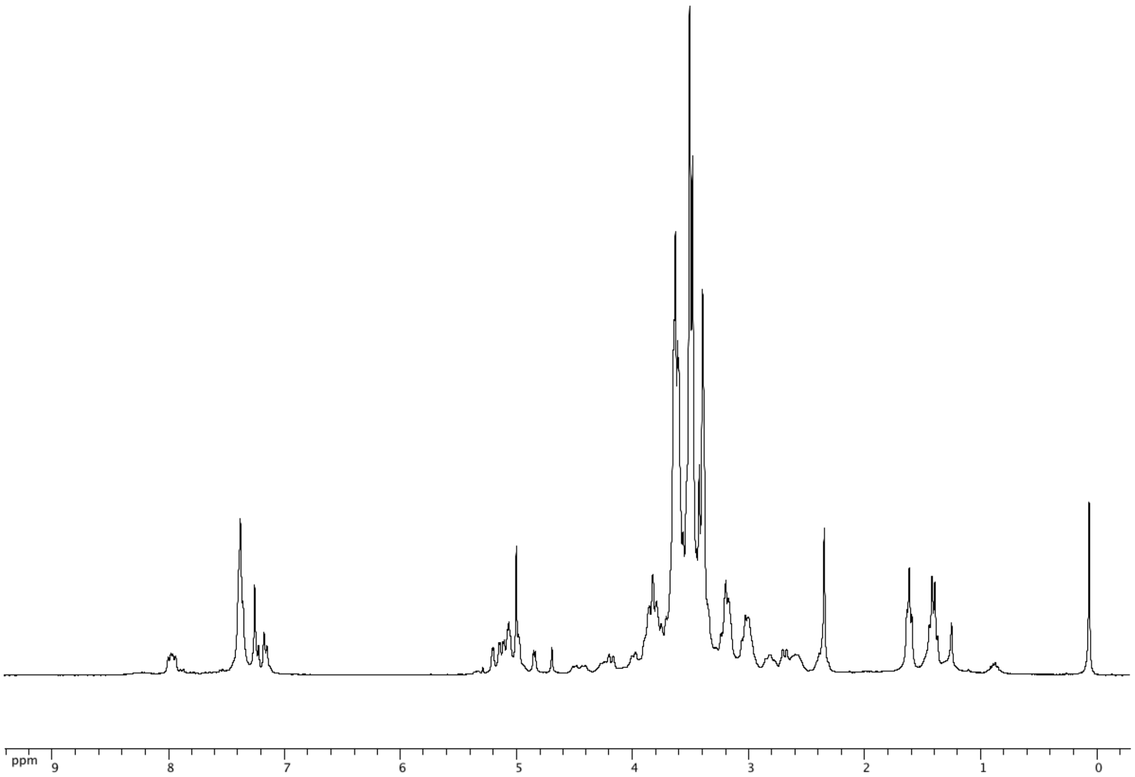

^{1}H NMR spectrum of **6** (300 MHz, $CDCl_3$, 25 °C)

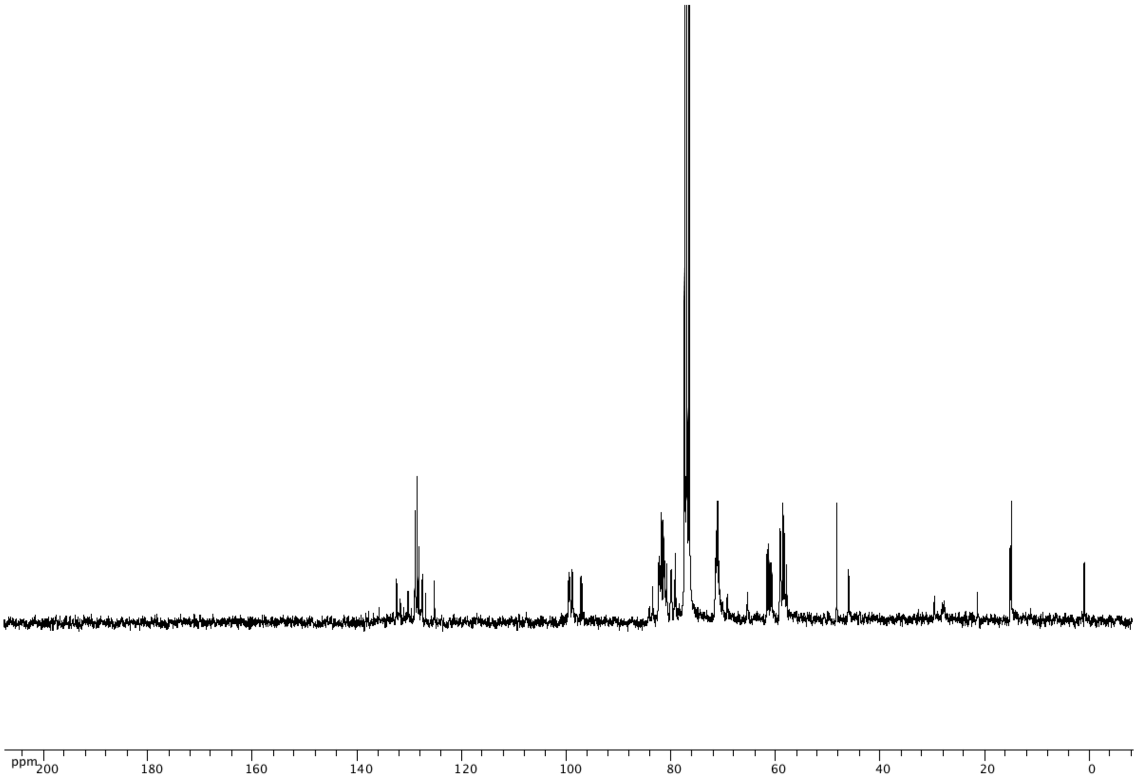

$^{13}C\{^{1}H\}$ NMR spectrum of **6** (75 MHz, $CDCl_3$, 25 °C)

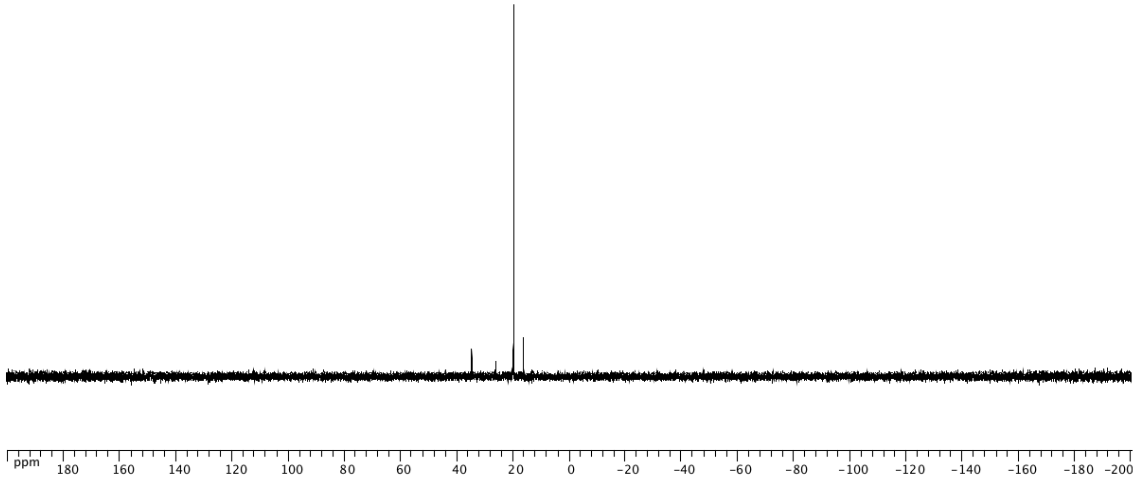

$^{31}P\{^{1}H\}$ NMR spectrum of **6** (160 MHz, $CDCl_3$, 25 °C)

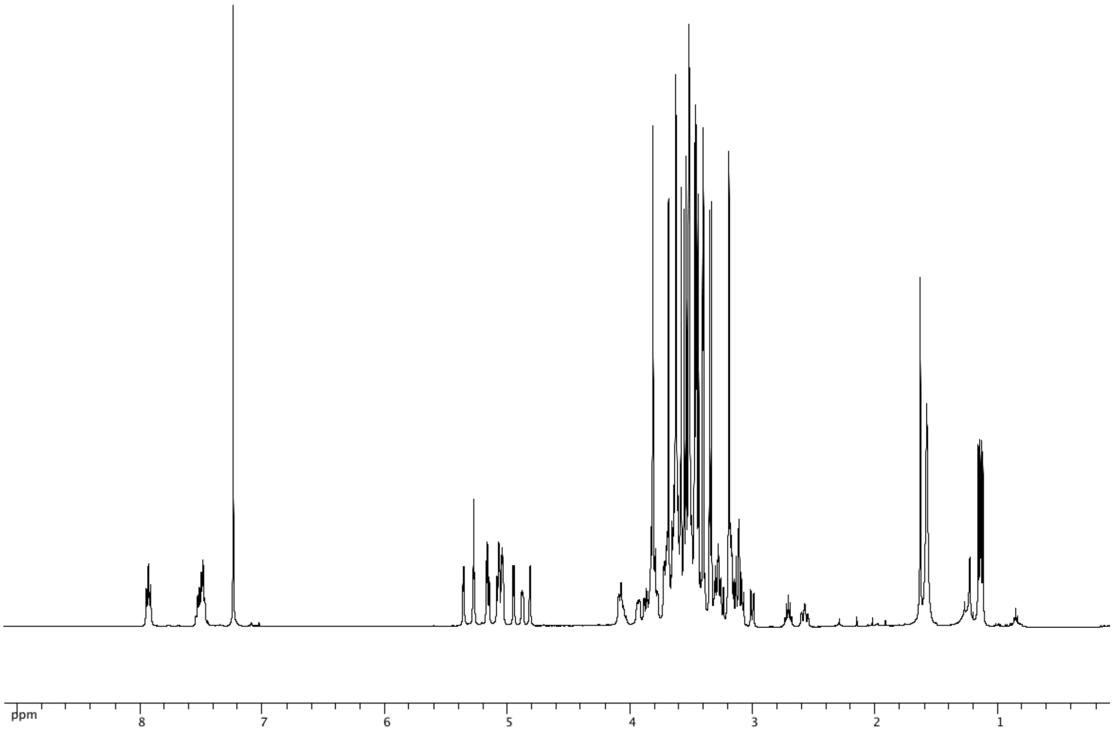

^{1}H NMR spectrum of **7** (500 MHz, $CDCl_3$, 25 °C)

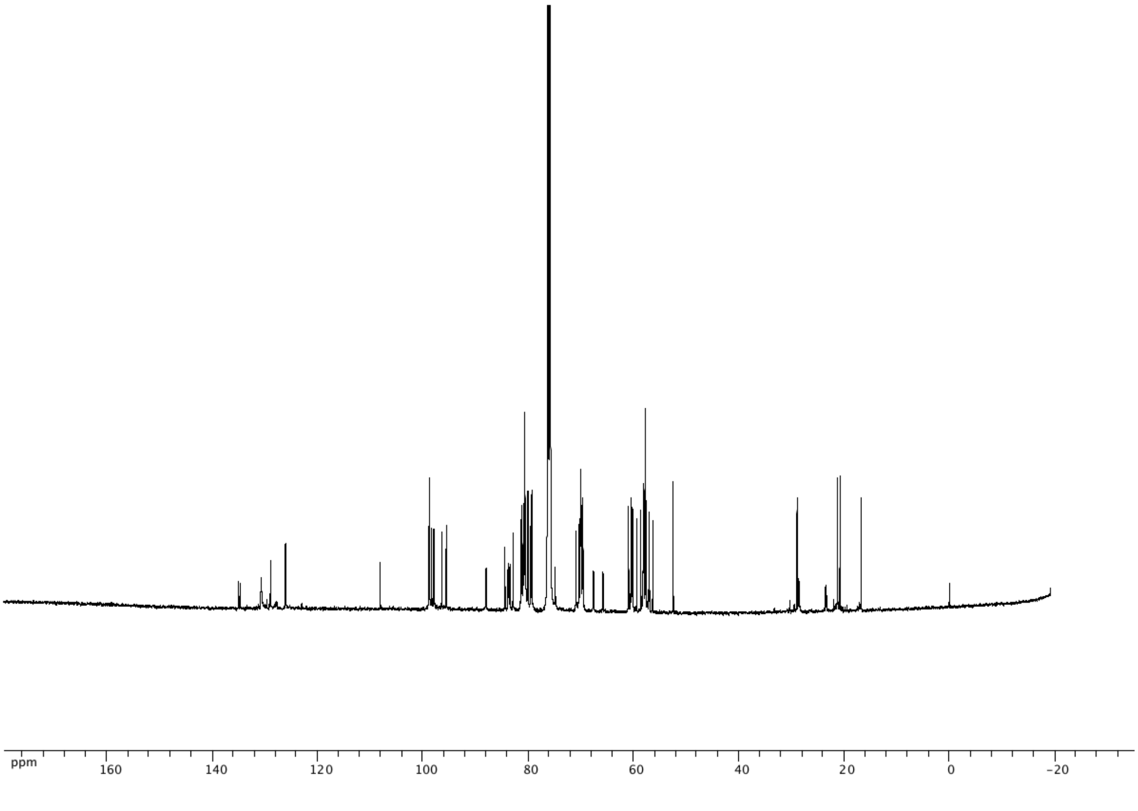

^{13}C{^{1}H} NMR spectrum of **7** (125 MHz, $CDCl_3$, 25 °C)

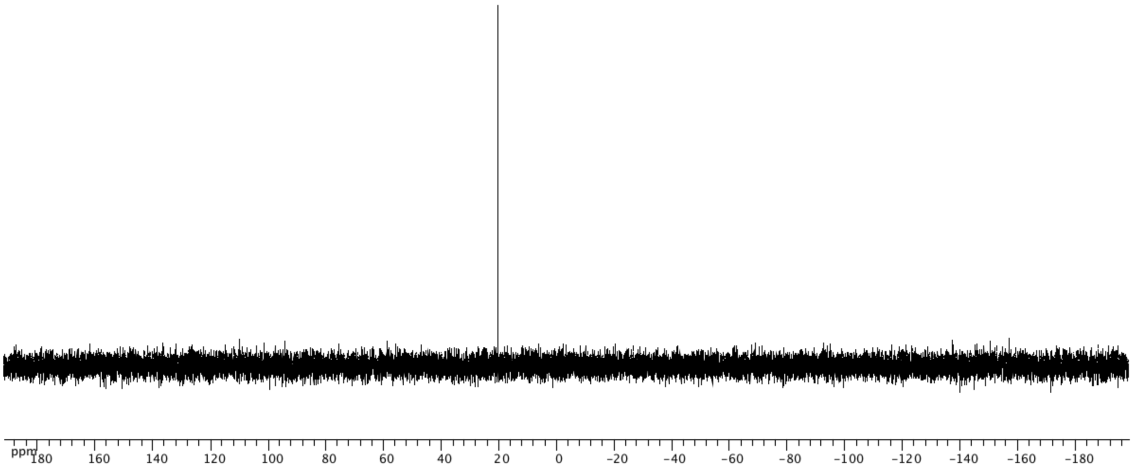

$^{31}P\{^{1}H\}$ NMR spectrum of **7** (160 MHz, $CDCl_3$, 25 °C)

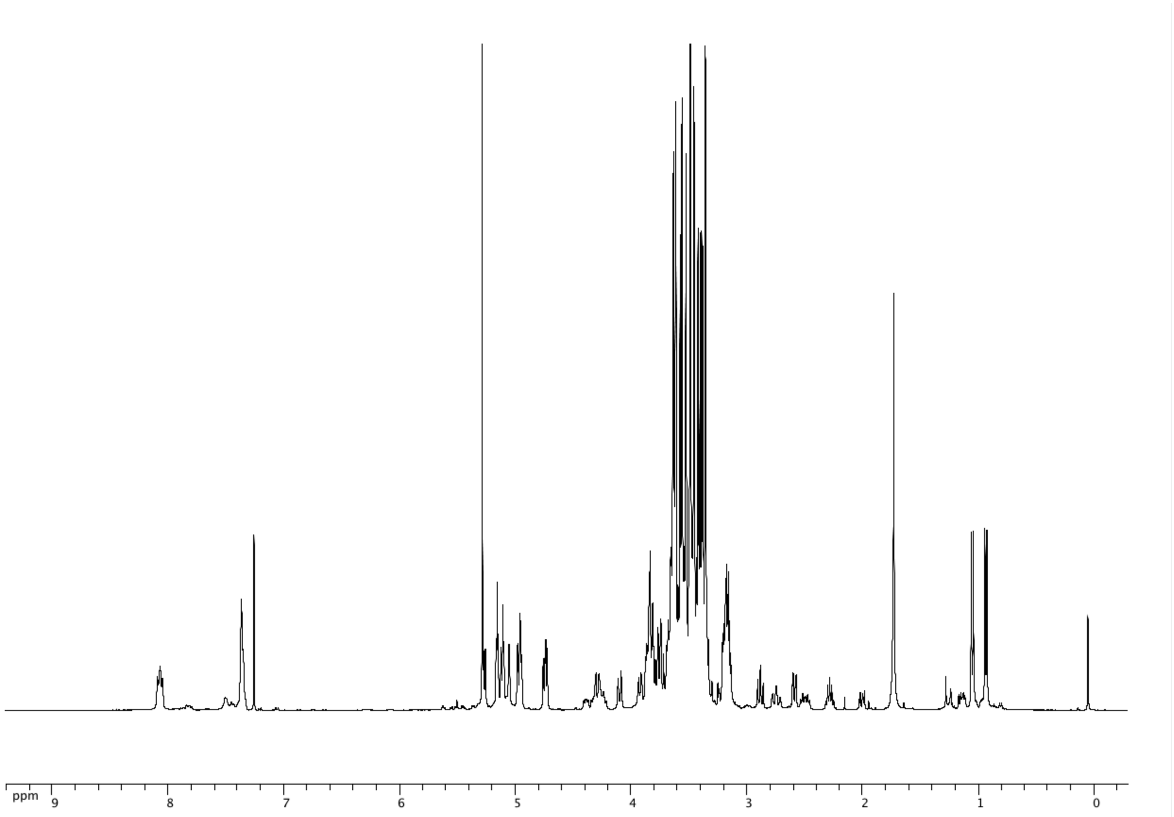

^{1}H NMR spectrum of **8** (400 MHz, $CDCl_3$, 25 °C)

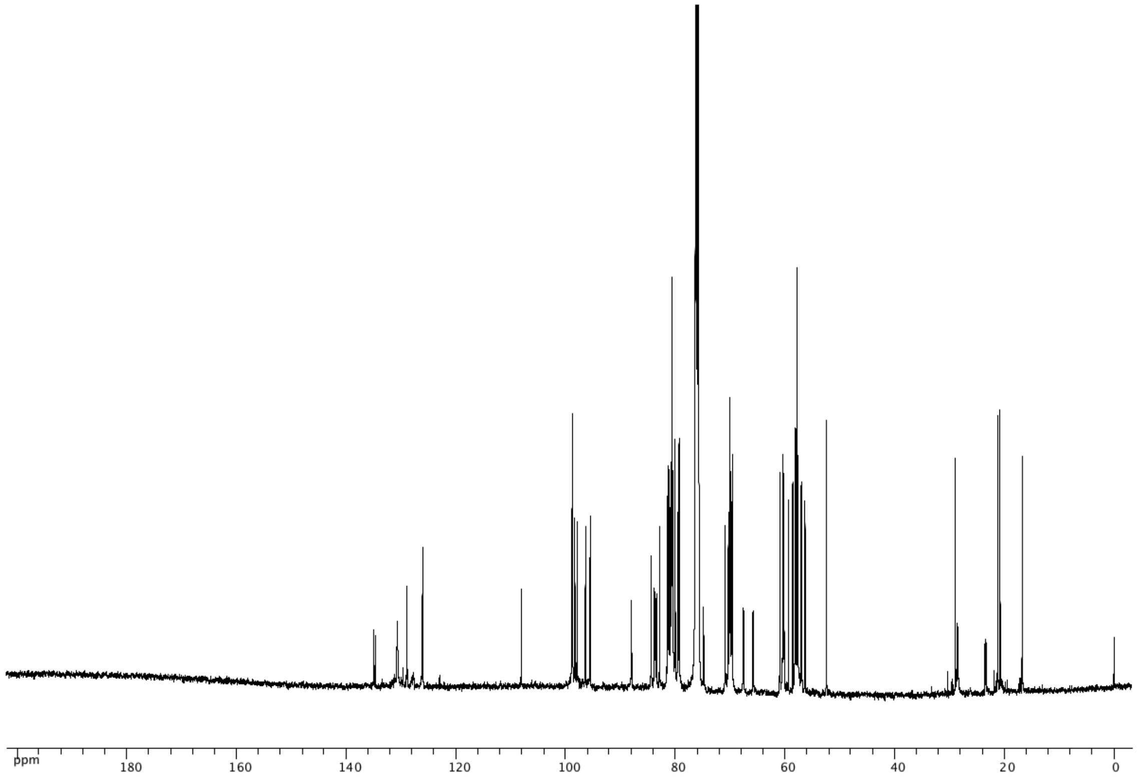

^{13}C{^{1}H} NMR spectrum of **8** (125 MHz, $CDCl_3$, 25 °C)

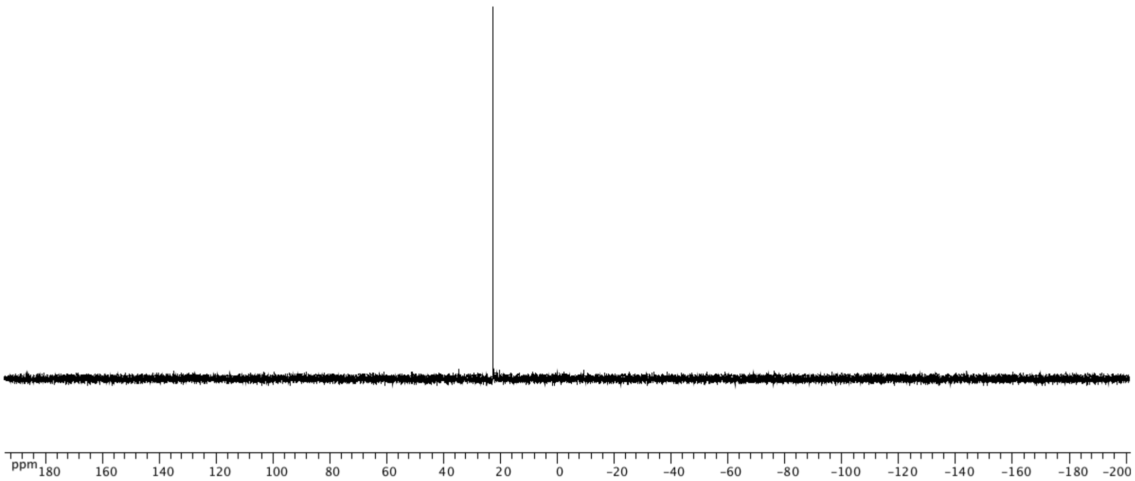

$^{31}P\{^{1}H\}$ NMR spectrum of **8** (160 MHz, $CDCl_3$, 25 °C)

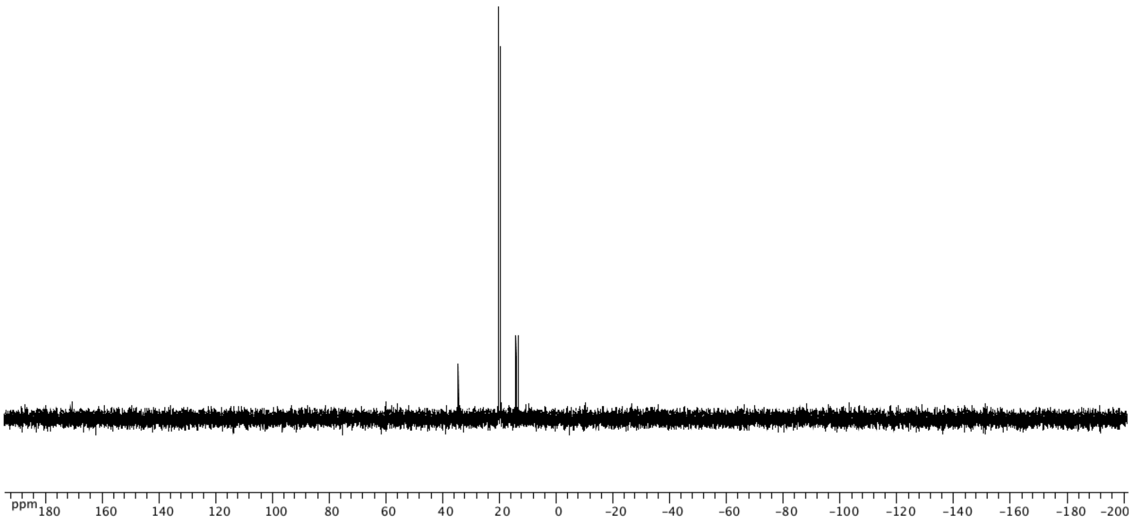

$^{31}P\{^{1}H\}$ NMR spectrum of **10a,b** (160 MHz, $CDCl_3$, 25 °C)

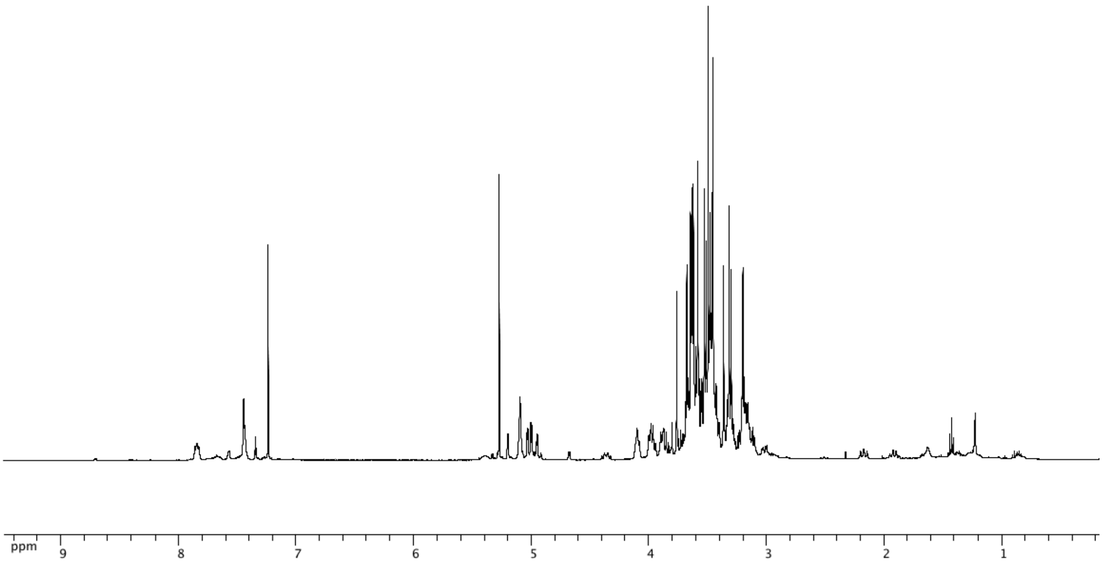

^{1}H NMR spectrum of **11** (500 MHz, $CDCl_3$, 25 °C)

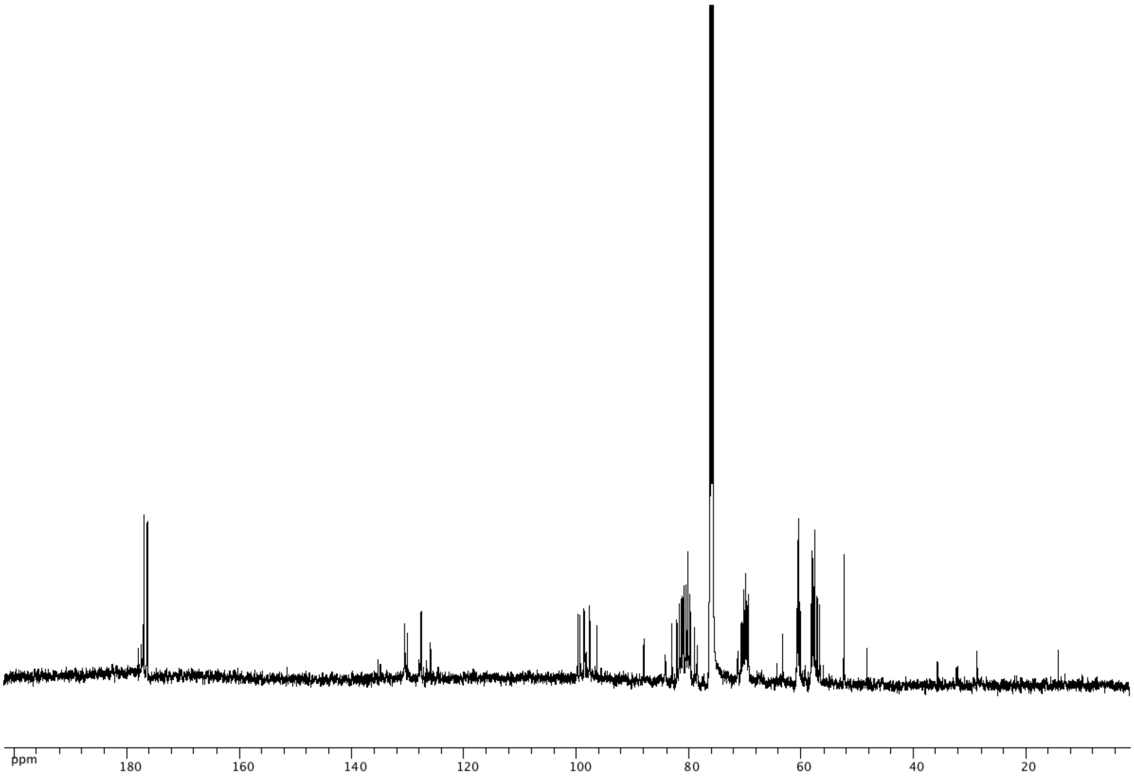

^{13}C{^{1}H} NMR spectrum of **11** (125 MHz, $CDCl_3$, 25 °C)

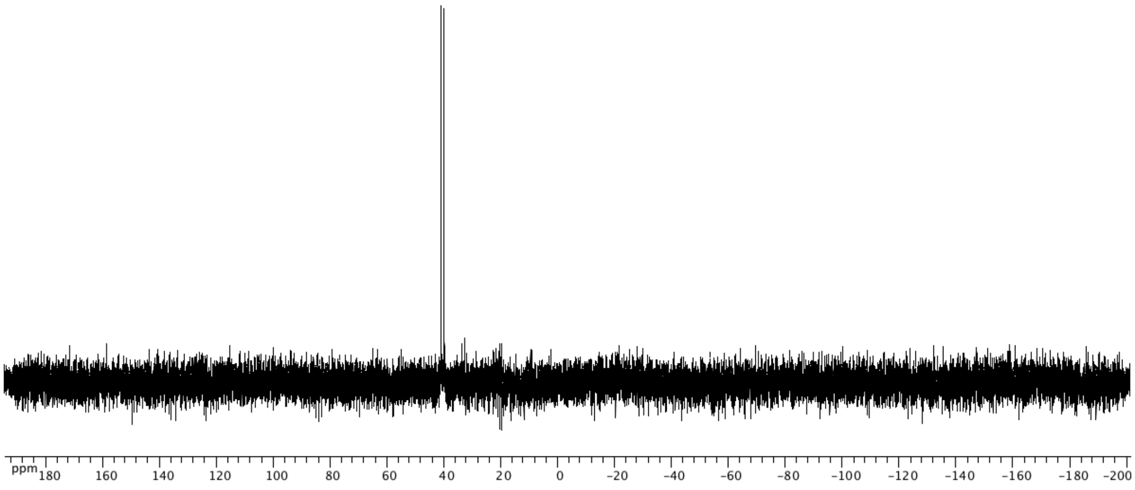

$^{31}P\{^1H\}$ NMR spectrum of **11** (160 MHz, $CDCl_3$, 25 °C)

General procedure for determining the glucose units linked by a given capping unit[1]

Our strategy for full structural assignment began with the differentiation between capped and non-capped C-6 carbon atoms by DEPT 135. These appear as two distinct sets of signals. The H-6 protons could then be identified using ^{1}H-^{13}C HMQC (Heteronuclear Multiple Qantum Coherence spectroscopy). By using TOCSY (TOtal Correlation SpectroscopY) and COSY (COrrelated SpectroscopY), each H-6 proton was correlated to the set of protons belonging to the same glucose residue. The connectivity between individual glucose units was then established via a ROESY (Rotating frame Overhauser Effect SpectroscopY) experiment showing the proximity between H-4_N and H-1_{N+1} protons (N and N+1 standing for neighbouring glucose moieties labelled in the alphabetical order).

References

[1] H.-J. Schneider, F. Hacket, V. Rüdiger, *Chem. Rev.* **1998**, *98*, 1755-1785.
